# A conserved guided entry of tail-anchored pathway is involved in the trafficking of a subset of membrane proteins in *Plasmodium falciparum*

**DOI:** 10.1371/journal.ppat.1009595

**Published:** 2021-11-15

**Authors:** Tarkeshwar Kumar, Satarupa Maitra, Abdur Rahman, Souvik Bhattacharjee

**Affiliations:** Special Centre for Molecular Medicine, Jawaharlal Nehru University, New Delhi, India; University of Geneva, SWITZERLAND

## Abstract

Tail-anchored (TA) proteins are defined by the absence of N-terminus signal sequence and the presence of a single transmembrane domain (TMD) proximal to their C-terminus. They play fundamental roles in cellular processes including vesicular trafficking, protein translocation and quality control. Some of the TA proteins are post-translationally integrated by the Guided Entry of TA (GET) pathway to the cellular membranes; with their N-terminus oriented towards the cytosol and C-terminus facing the organellar lumen. The TA repertoire and the GET machinery have been extensively characterized in the yeast and mammalian systems, however, they remain elusive in the human malaria parasite *Plasmodium falciparum*. In this study, we bioinformatically predicted a total of 63 TA proteins in the *P*. *falciparum* proteome and revealed the association of a subset with the *P*. *falciparum* homolog of Get3 (PfGet3). In addition, our proximity labelling studies either definitively identified or shortlisted the other eligible GET constituents, and our *in vitro* association studies validated associations between PfGet3 and the corresponding homologs of Get4 and Get2 in *P*. *falciparum*. Collectively, this study reveals the presence of proteins with hallmark TA signatures and the involvement of evolutionary conserved GET trafficking pathway for their targeted delivery within the parasite.

## Author summary

Tail-anchored (TA) membrane proteins are known to play essential cellular functions in the eukaryotes. These proteins are trafficked to their respective destinations by post-translational translocation pathways that are evolutionarily conserved from yeast to human. However, they remain unidentified in the malaria parasite *Plasmodium falciparum*. We have used bioinformatic prediction algorithms in conjunction with functional validation studies to identify the candidate TA repertoire and some of the homologs of the trafficking machinery in *P*. *falciparum*. Initially, we predicted the presence of 63 putative TA proteins localized to distinct compartments within this parasite, including a few confirmed TA homologs in other eukaryotic systems. We then identified and characterized PfGet3 as a central component in the Guided-Entry of TA (GET) translocation machinery, and our bacterial co-expression and pulldown assays with two selected recombinant TA proteins, PfBOS1 and PfUSE1, showed co-association with PfGet3. We also identified PfGet2 and PfGet4 as the other two components of the GET machinery in *P*. *falciparum* using proximity biotinylation followed by mass spectrometry. Interestingly, we also found six TA proteins in the parasite enriched in this fraction. We further validated the direct interactions between a few TA candidates, PfGet4 and PfGet2 with PfGet3 using recombinant-based pulldown studies. In conclusion, this study classified a subset of membrane proteins with the TA nomenclature and implicated a previously unidentified GET pathway for their translocation in this apicomplexan parasite.

## Introduction

Integral membrane proteins constitute ~20–30% of the total eukaryotic proteome where they serve essential cellular functions including vesicular sorting, solute transport, protein homeostasis and organelle biosynthesis. Thus, precise targeting of membrane proteins to their respective subcellular destinations is often dictated by the evolutionary conserved and sophisticated trafficking mechanisms. Most membrane proteins are inserted through the chaperone-assisted and co-translational pathway, which involves recognition of ribosome-associated nascent chains (RNC) by the signal recognition particle (SRP), targeting to the SRP-receptor at the ER membrane, and their release to the Sec61 translocon [1–4]. The Sec61 complex subsequently facilitates TMD integration into the lipid bilayer as they emerge out from the ribosomes [5–8]. The major advantage for the co-translational targeting is a tightly coordinated relay of events between the protein synthesis, targeting and membrane insertion to ensure efficient shielding of the hydrophobic TMDs from the bulk hydrophilic cytosolic milieu. However, not all membrane proteins recruit the SRP/Sec61 route for insertion. Tail-anchored (TA) proteins are one such unique class of integral membrane proteins characterized by the absence of any N-terminus signal sequence (SS) and the presence of a single helical transmembrane domain (TMD) at or near their C-terminus (CTS) [9]. This close proximity of the TMDs in TA proteins places it within the ribosomal tunnel, thus precluding SRP/Sec61-mediated co-translational insertion; and consequently, TA proteins must target in a strictly post-translational manner [10–12]. Notable examples include proteins of the vesicular trafficking pathway (the SNAREs, Soluble NSF Attachment protein REceptors), ER and mitochondrial subunit translocation machinery, mitochondrial electron carrier (cytochrome b5/Cb5) and outer mitochondrial membrane proteins that regulate apoptosis (Bcl family) or mitochondrial dynamics (*e*.*g*., Fission 1/FIS1) (reviewed in [13]). TA proteins have been identified across evolutionary diverse organisms, including *Saccharomyces cerevisiae*, bacteria, *Homo sapiens*, *Arabidopsis thaliana*, and more recently, in the apicomplexan parasite *Toxoplasma gondii* [14–18]. The TA biogenesis is well-characterized for proteins localized specifically to the ER and then transported from their ER-integrated state to the other cellular compartments (such as plasma membrane, nuclear envelope, Golgi complex, endosomes, lysosomes, and peroxisomes) *via* the network of secretory vesicles (reviewed in [19, 20]). Multiple pathways are implicated in the targeting of TA proteins destined for the ER, including a promiscuous ‘moonlighting function’ by the SRP/Sec61 translocon (also involved in the co-translation translocation) [21, 22], insertion mediated by the SRP-independent targeting (SND) components [23], non-assisted delivery and insertion of TA proteins regulated by the cytosolic factors [24] and the components of the ER membrane complex (EMC) [25, 26]. The TA proteins with more hydrophobic TMDs (compared to the mildly hydrophobic TMD of Cb5, which inserts in an unassisted manner) require the assistance of chaperones from the Hsp40/Hsc70 family [27, 28]. Thus, targeting information for the sorting of nascent TA proteins is not defined by the presence of specific sequence motif within their TMDs, rather it relies on physicochemical properties such as the length, hydrophobicity index and charge of the TMDs, as well as the length and the net charge of the CTS. For example, as compared to ER-localized TA proteins, mitochondrial-destined TA proteins tend to possess TMDs that are shorter and less hydrophobic, and their CTSs are enriched in positively charged amino acid residues [13].

In the yeast and mammalian cells, a subset of the TA protein repertoire destined for the ER (or post-ER compartments) rely on a proteinaceous machinery known as the Guided Entry of TA (GET) or transmembrane domain recognition complex (TRC), respectively (reviewed in [29, 30]). However, intersectional competition between the SRP and the GET targeting pathways for the same RNCs and their convergence at the Sec61 translocon has also been observed [31]. In the GET pathway, the ER-destined TA proteins are first recognized by a small glutamine-rich tetratricopeptide repeat (TPR)-containing protein Sgt2 through the C-terminus hydrophobic binding domains and then transferred to ER-targeting cytosolic ATPase Get3, facilitated by the interactions with Get4/5 complex [32]. Molecular chaperones of the Hsp70 family are also implicated to function upstream of Sgt2 in some studies [33, 34]. The TA-loaded Get3 subsequently interacts with the ER-resident Get1/2 transmembrane receptors that culminates in active membrane integration of the TA cargo in an ATP-dependent manner. In contrast, the TA insertion events in mammalian cells are more complex; in addition to homologs of Sgt2 (small glutamine-rich TPR-containing α; SGTA), Get4 (TRC35), Get5 (ubiquitin-like protein 4A; Ubl4A), Get3 (TRC40) and Get1/2 (tryptophan rich basic protein/calcium-modulating cyclophilin ligand; WRB/CAML), higher eukaryotes also involve mediation by Bag6 (also known as BAT3/Scythe) in a complex together with TRC35 and Ubl4A (constituting the Bag6 complex) that facilitates substrate transfer from SGTA to TRC40 [35]. Since Bag6 is primarily involved in the ubiquitination of defective ribosomal proteins or mislocalized secretory and membrane proteins, its presence likely confers a protein quality control aspect to the TRC pathway. Interestingly, binding of substrate proteins to SGTA has been shown to revert Bag6-mediated ubiquitination and promote deubiquitination of mislocalized proteins, essentially shunting away TA clients for their delivery to the ER and not for their degradation [36–38].

In a recent study, 59 novel TA proteins were identified in a related apicomplexan parasite *T*. *gondii* [18]. Domain swapping experiments further suggested an interplay between the sequence of the TMD and charge distribution in the CTS as deciding factors for the TA distribution across the ER, mitochondria, and the Golgi apparatus. However, neither TA proteins nor the homologs of the GET/TRC pathway have ever been theoretically identified or experimentally validated in the human malaria parasite *Plasmodium falciparum*. *P*. *falciparum* causes the most severe form of human malaria [39]. During its asexual stages, the parasite invades and proliferates within human erythrocytes from early ring stages to mature trophozoites, and finally schizonts undergoing schizogony to form differentiated segmenters, which then liberate daughter merozoites in the blood stream after the erythrocytic rupture. Although the terminally differentiated red cells ensure a safe haven for the parasite away from the host immune system, they lack optimal infrastructure conducive for its nutrient and secretory needs. So, *P*. *falciparum* develops a complex network of membranes and vesicles for efficient protein trafficking [40]. These events help the parasite in its *in vivo* survival and ensure that the virulence determinants are trafficked correctly to their destined organelles. Protein trafficking in malaria-infected erythrocytes requires an additional level of refinement since the parasite resides within a parasitophorous vacuole (PV) of its host and can target proteins not only to the intra-parasite organelles but further beyond, to the parasitophorous vacuolar membrane (PVM) and the infection-induced modified host erythrocytic structures (such as Maurer’s clefts, erythrocyte cytosol and red cell membrane knobs) [41] (reviewed in [42]). The early secretory system in *P*. *falciparum* necessarily recruits the function of the SS similar to the higher eukaryotes [43]. However, host-exported proteins require the presence of an additional leader sequence, known as *Plasmodium* export element (PEXEL) or host-targeting (HT) motif with a consensus RxLxE/D/Q (where x is any amino acid) [44, 45], and their export involves a multiprotein translocon complex [46]. Nonetheless, HT-independent trafficking has since been reported [47] and PEXEL-negative exported proteins (PNEPs), lacking any discernible export motif are also found in the infected erythrocyte membranes [40]. In *P*. *falciparum*, the parasite ER functions as an obligatory destination for proteins during the initial stages of the secretory pathway. At the ER, important sorting decisions are made and exported proteins reportedly bind phosphatidylinositol 3-phosphate-enriched domains through their HT/PEXEL motif [48] prior to their cleavage by the ER-resident aspartic protease plasmepsin V [49, 50]. However, the export pathway (*via* ER-recruitment) need not necessarily involve SS since PNEPs without any N-terminus signal peptide are efficiently recruited and exported to the Maurer’s clefts and to the host erythrocyte [51].

Irrespective of the route of trafficking or the final locale within an infected erythrocyte, the roles of vesicular transport intermediates as carriers of virulence determinants are undisputed in *P*. *falciparum* (reviewed in [52]). Model TA proteins, validated in other systems such as Syntaxins, SNAREs and NSF, are implicated in directing the budding and fusion of secretory vesicles in *P*. *falciparum*. Thus, there is an urgent need to identify the TA proteins and the underlying GET/TRC pathway in *P*. *falciparum*. In this study, we *firstly* identified a *P*. *falciparum* homolog of Get3 (PfGet3) based on the sequence, predicted structural, and functional similarity to the Get3s of prokaryotic and eukaryotic origins. *Secondly*, we showed that PfGet3 associates with a subset of the predicted total 63 putative TA proteins in the *P*. *falciparum* proteome. Our proximity labelling experiments further revealed the identities of the two other homologs of the GET machinery in *P*. *falciparum*, such as PfGet4 and PfGet2, and we validated their association with PfGet3 by *in vitro* binding studies. We were, however, unable to definitively identify the plasmodial homologs of other GET components, such SGTA/Sgt2, Get5/UBL4A and Bag6 from our shortlisted proteins since all of them featured either the TPR motif (in the case of SGTA/Sgt2 homologs) or the characteristic ubiquitin-like domain (UBL; for Get5/Ubl4A and Bag6), both of which are ubiquitously present across multiple proteins enriched from our proximity labelling studies and LC-MS/MS analyses. We were also unable to identify the plasmodial homolog of Get1/WRB due to extreme sequence divergence. Nonetheless, we believe this is the first study that provides bioinformatic and biochemical evidence for the presence of TA proteins and the partners of the GET machinery in the human malaria parasite *P*. *falciparum* and has the potential to serve as a leading resource for future investigations elucidating the dynamics of this pathway and its development for therapeutic interventions.

## Results and discussion

### A repertoire of tail-anchored (TA) proteins in the *P*. *falciparum* proteome

**Fig 1:**
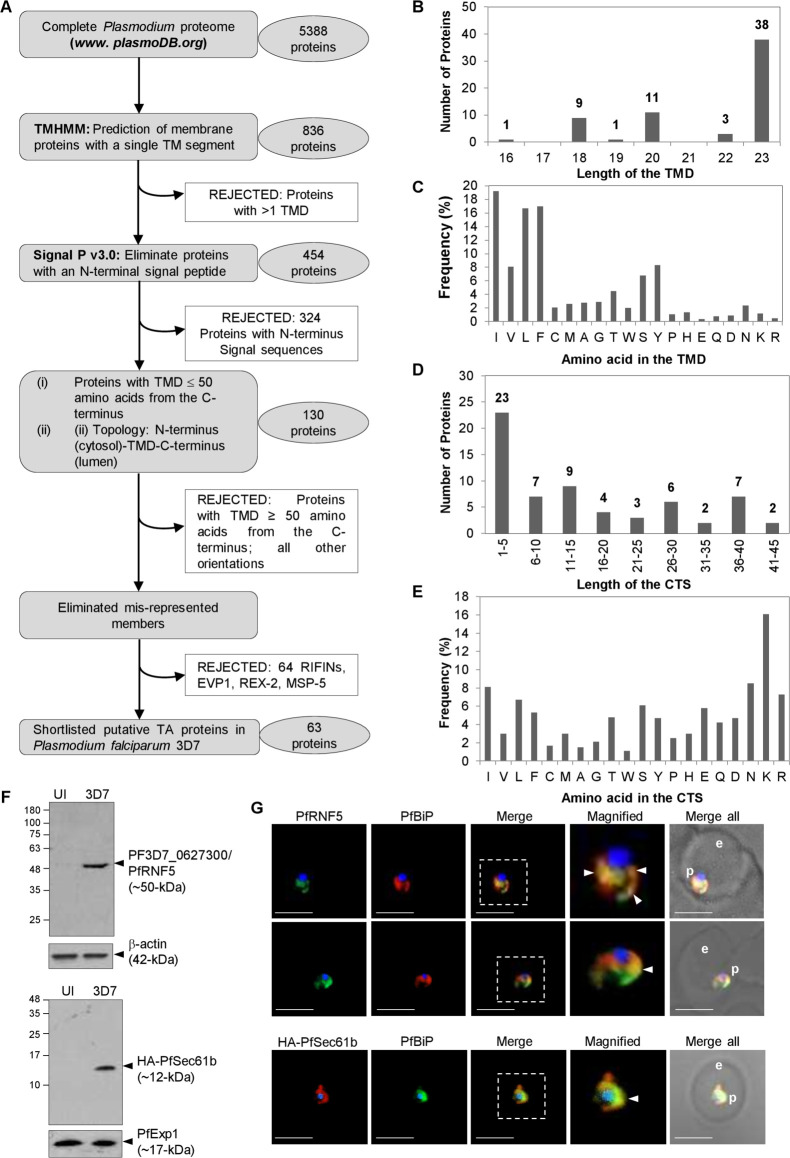
Identification of putative Tail-Anchored (TA) proteins in *P*. *falciparum* 3D7. **A.** Flow diagram showing the successive bioinformatic steps and the final outcome for the 63 predicted TA proteins in *P*. *falciparum* 3D7. **B-C.** Lengths of the predicted TMDs as according to TMHMM (*www.cbs.dtu.dk/services/TMHMM*) (B) and their amino acid compositions (C) for the 63 TA proteins. **D-E.** Lengths of the CTSs (D) and the amino acid compositions (E). The number of predicted TA proteins are shown above the range of the TMD (B) and CTS (D) lengths. Amino acids listed (in C and E) are in decreasing order of hydrophobicity according to the Kyte and Doolittle scale [137]. **F.** Western blots showing the detection of PfRNF5 at ~50-kDa (arrowhead, top panel) by the commercial antibodies to human RNF5 (ABclonal, USA; AB8351) in infected erythrocytes (3D7, right lane) but not in uninfected (UI, left lane) red blood cells. The bottom panel shows western blot detecting the expression of HA-PfSec61b in transgenic parasites (right lane) but not in the parental 3D7 (left lane). Antibodies to the human β-actin (42-kDa; top panel) or the parasite Exported protein-1 (PfExp-1; ~17-kDa; bottom panel) are used as respective loading controls. Molecular weight standards (in kDa) are as indicated. **G.** IFA images showing regions of colocalization (arrowheads) between PfRNF5 (top and middle panels; green) in *P*. *falciparum* 3D7 or HA-PfSec61b transgenic parasites (bottom panel; red) with the ER marker PfBiP (red in the top and the middle panels; green in the bottom panel). Corresponding fluorescent and merged (with brightfield) images are as shown. Boxed areas (dotted outline) are magnified at the right. For all IFA images, scale bar, 5 μm; e, erythrocyte; p, parasite.

To date, no annotation as designated ‘TA protein’ exist in the *P*. *falciparum* database. We thus used sequential selection criteria in the PlasmoDB (*www*.*plasmodb*.*org*) to identify putative TA proteins in the *P*. *falciparum* 3D7 strain, based on the following classical TA features: (i) the absence of an ER-type secretory SS as predicted by SignalP (*http*:*//www*.*cbs*.*dtu*.*dk/services/SignalP*), (ii) the presence of a single TMD within 50 amino acids from the C-terminal end of the protein, and (iii) the orientation of the protein with respect to the TMD, *i*.*e*., we favoured N-terminus_cytosolic_-TMD-C-terminus_lumenal_ orientation (using the TMHMM server at *http*:*//www*.*cbs*.*dtu*.*dk/services/TMHMM*) (the schematic flow chart shown in **Fig 1A**). The output revealed a total of 130 proteins with these signatures (**S1 Table)**, including homologs previously validated as TA proteins in other systems, such as Sec61β (PF3D7_0821800), guanine-nucleotide exchange factor Sec12 (PF3D7_1116400), SNARE proteins like BOS1 (PF3D7_1111300), VAMP7 (PF3D7_0826600), VAMP8 (PF3D7_1303200), mitochondrial Fission1 protein FIS1 (PF3D7_1325600) (a representative subset is also shown in **Table 1**). We manually removed the 67 misrepresented members from the list of outputs based on results from the earlier studies. These included 64 members of the repeated interspersed multigene family (RIFINs), mainly the subgroup RIFIN-A implying them as putative TA proteins. RIFINs constitute the largest known family of variant antigens in *P*. *falciparum* (~150 copies per haploid 3D7 genome) and encode for 27-45-kDa proteins [53, 54]. Previous studies support the absence of a SS and the presence of a single TMD in RIFIN-As, while RIFIN-Bs are assumed to possess SS and two TMDs [55]. Members of RIFIN-A possess HT/PEXEL motif and are trafficked to the infected erythrocyte membrane *via* the parasite’s induced structures called Maurer’s clefts [56]. Although we cannot comprehensively rule out the possibility that the members of RIFIN-A may function as TAs and follow the specific TA-trafficking route at their initial translocation event, they were excluded in this study solely based on their previous localization reports. Similarly, two other parasite proteins, *i*.*e*., PF3D7_0410000 (erythrocyte vesicle protein-1) and PF3D7_0936000 (ring-exported protein-2) were already confirmed to be exported to the host erythrocytes [57, 58] and were also eliminated since TA proteins are generally perceived to be confined within the cellular boundary. In addition, PF3D7_0206900 (merozoite surface protein 5), a GPI anchored protein [59] was also removed from the TA list (see **S1 Table** for the full list of eliminated proteins). The final outcome from our predictions enlisted a total of 63 TA proteins in *P*. *falciparum* 3D7 (**Table 1**; full list is displayed in **S1 Table**).

**Table 1:** A representative list of the predicted TA proteins in *P*. *falciparum* 3D7. Proteins are listed according to their PlasmoDB ID (*https://plasmodb.org*) and descriptions. Attributes of each protein include the transmembrane domain (TMD) sequence and its calculated hydrophobicity score (according to GRAVY calculator; *http://www.gravy-calculator.de*), the C-terminus sequence (CTS) and the net charge of the CTS. Predicted organellar localizations, as designated in this study, are also indicated. The complete list of TA proteins is shown in **S1** *Table*.

Gene ID	Description	TMD sequence	TMD hydrophobicity (GRAVY score)	CTS sequence	Net charge (CTS)	Predicted localization (this study)
PF3D7_0821800	SEC61β; Protein transport protein	LTPQTVLISTLIFMASVVILHII	1.94	SKI	+1	ER
PF3D7_1116400	SEC12; guanine-nucleotide exchange factor	FIIYFLLLFFISIILLDSFNVGY	2.09	DLRISSLFHNKAYTNITKKKRNYNVDPKKNKVIMDMDEL	+4.1	ER
PF3D7_1111300	BOS1, SNARE protein, putative (GS27)	NLIIVIVGIILSLIFFYVIYSYF	2.36	KR	+2	ER
PF3D7_0826600	VAMP7; SNARE protein	YWKTYFLCACFLIIGFKVY	1.17	RSI	+1	ER
PF3D7_1303200	VAMP8; SNARE protein	MTLYFISSIIIFIFFIWSLY	2.05	NV	0	ER
PF3D7_0217300	AP-2 complex subunit sigma	LFNFHFLYYFFDNIILGGYIYEI	0.87	NRNIILDKINKIKKLI	+4	Mitochondria
PF3D7_1325600	Mitochondrial fission 1 protein, putative (FIS1)	ISSDGLIGALLVALTACGLYLSF	1.63	KSFKYF	+2	Mitochondria
PF3D7_0806300	Ferlin, putative	WTGVWIVVGVIVIGIFFLIFLF	2.66	K	+1	Other

The TA proteins enter multiple cellular membranes including the endomembranous ER, Golgi, plasma membrane, mitochondria, and peroxisomes (and chloroplasts, in case of plants) (reviewed in [19, 20, 60]). The lengths of the TMDs and the amino acid composition of the CTS play important roles in the organellar localization of TA proteins across different species [61–63]. Mitochondrial TA proteins often display significantly lower TMD hydrophobicity compared to the ER-destined pool [13]. A recent study carefully dissected the TMD and the CTS of multiple TA proteins in mammalian cells and implicated a delicate balance between the extent of TMD hydrophobicity and the net charge of the CTS in deciding the TA trafficking and/or their sharing between different organelles [64]. Peroxisomal TA proteins were shown to possess significantly higher net positively charged CTS as compared to those routed to the mitochondria, and the ER-destined TA proteins scored the least positively charged CTS. We thus investigated these features across the 63 putative TA proteins in *P*. *falciparum* 3D7. The TMD lengths varied between 18 to 23 amino acids among the TA proteins (TMHMM *v*.*2*.*0*; *http://www.cbs.dtu.dk/services/TMHMM*), with an average length of 22 amino acids, correlating well within the helical length of 18–21 amino acids necessary to span the usual width of a lipid bilayer (**Fig 1B**). A bias towards the enrichment of amino acids with hydrophobic side chains was also observed across these TMDs (**Fig 1C**). In contrast, the length of the CTS was diverse, varying from 1–5 amino acid (23 putative TA proteins) to as high as 45 amino acids (in PF3D7_1422000) (**Fig 1D**). Also, there was no clear preference for any particular type of amino acids in the CTS (**Fig 1E**). In the GRAVY scale (*described in the* ***S1 Text***), the TMD hydrophobicity values ranged from the lowest 0.74 in PF3D7_1422000 to the highest 3.45 in PF3D7_1341700. PF3D7_1422000 is annotated as single pass putative cytochrome c oxidase assembly protein (COX14) that localizes to the mitochondria [65]. PF3D7_1341700, on the other hand, is a conserved plasmodial protein with unknown function. The other TA proteins with low hydrophobicity include PF3D7_0217300 (Adaptor protein complex-2 subunit σ; GRAVY score 0.87), playing an active role in vesicular membrane protein transport [66], and PF3D7_0306000 (putative cytochrome b-c1 complex subunit 8; GRAVY score 0.91) which is part of the mitochondrial electron transport chain driving oxidative phosphorylation [67]. Similarly, other annotated TA proteins with high hydrophobicity indices include PF3D7_1332000 (Syntaxin 5; GRAVY score 3.04), PF3D7_0210700 (Syntaxin 17; GRAVY score 2.88) and PF3D7_0710800 (putative protein transport protein, USE1; GRAVY score 2.92) which constitute the SNARE complex and mediates anterograde or retrograde transport between ER and Golgi (reviewed in [68]). The ER-specific TA proteins identified in our list include PF3D7_0821800 (Protein transport protein Sec61 subunit β) and PF3D7_1116400 (Guanine nucleotide-exchange factor SEC12) and others, with an intermediate GRAVY score of 1.94 and 2.09, respectively. Similarly, lower Agadir scores are typically associated with TA proteins targeted to the mitochondria (mitochondrial fission 1 protein FIS1, PF3D7_1325600; Adagir score 1.39), while higher Agadir scores represent TA proteins targeted to the ER (syntaxin SYN11, PF3D7_1432000; Adagir score 10.52) [69].

We also manually grouped the 63 putative *P*. *falciparum* TA proteins into three broadly predicted sub-cellular localizations; namely the ER (and Golgi), the mitochondria and a collective of other destinations (including the endosomes/lysosomes, plasma membrane, peroxisomes, chloroplast etc.), based on the Gene Ontology (GO) annotations in the Uniprot database (*http*:*//uniprot*.*org*) (*see* ***S1 Text*** *for the predicted distribution criteria details*). Our analyses revealed a total of 14 ER-specific TA proteins, 7 mitochondrial-specific TAs and 42 TAs with diverse predicted cellular destinations (**S1 Table)**. The distribution profiles for some of the predicted TA proteins in *P*. *falciparum* or their corresponding apicomplexan homologs have been previously reported in other studies. These include PF3D7_1332000/PfSyn5) and PF3D7_1111300/BOS1/GS27), primarily localized to the ER-derived vesicles and *cis*-Golgi in *P*. *falciparum* and *T*. *gondii* [70, 71]; the malarial homolog of the eukaryotic FIS1 (PfFIS1/PF3D7_1325600) localized to the parasite mitochondrion, and the *P*. *berghei* homolog of Sec61b localized to the parasite ER and exhibited high degree of co-localization with the ER resident *P*. *falciparum* Binding Protein (PfBiP) [72]. These were consistent with the organellar localization predicted by us. We further analyzed the intracellular locales of PfRNF5 (PF3D7_0627300) and PfSec61b (PF3D7_0821800). A commercial antibody, anti-RNF5 (ABclonal Inc., USA; Catalog no. A8351) generated using a recombinant fragment of the *H*. *sapiens* RNF5 (a TA homolog sharing significant identity to the plasmodial PfRNF5), selectively identified PfRNF5 at ~50-kDa (calculated molecular weight 52812 Da) in the infected erythrocytic fraction by western blot (**Fig 1F**, top blot). In IFAs, PfRNF5 also showed distribution at the perinuclear ER compartments and some degree of co-localization with PfBiP (**Fig 1G**, top and middle panels), consistent with the human RNF5 [73] Similarly, we generated transgenic parasites expressing HA tagged to PfSec61b at the N-terminus. Antibodies to the HA tag recognized HA-PfSec61b at ~12-kDa in the transgenic 3D7 by western blotting (**Fig 1F,** bottom blot). HA-Sec61b also exhibited ER distribution with good colocalization with PfBiP (**Fig 1G**, bottom panel). Both PfRNF5 and PfSec61b have GRAVY scores of 1.87 and 1.94, respectively and with short CTS (2 or 3 amino acids) and 0/1 net charge. These have been predicted as ER/Golgi localized proteins (**S1 Table**).

Thus, the diverse features exhibited by the TMD and CTS of the predicted TA proteins in *P*. *falciparum* and the outcome of our cellular localization assays for a few candidate TAs were consistent with the characteristics exhibited by TA protein in the other systems, thereby validating our bioinformatic selection procedures (a few representative TA proteins are shown in **Table 1**).

### Identification of a putative homolog of Get3 in *P*. *falciparum*

**Fig 2:**
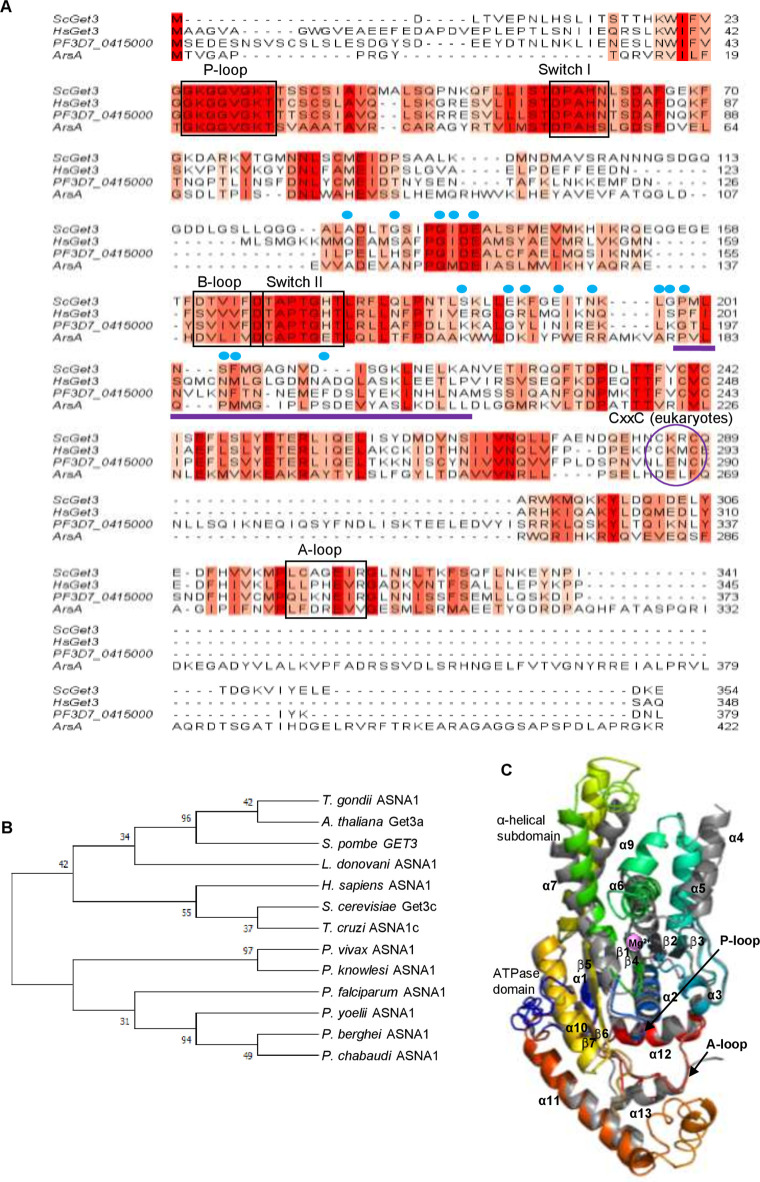
Identification and characterization of a *P*. *falciparum* homolog of Get3. **A.** Sequence alignments between the three eukaryotic (PF3D7_0415000/PfGet3, ScGet3, HsGet3) and one prokaryotic (ArsA) Get3 homologs highlighted in the different shades of red based on their conservation. The four ATPase sequence motifs are in boxed rectangles and the zinc-binding ‘ÇxxC’ motif is boxed oval. The ~20 residue Get3/TRC40 insert is underlined in purple, and the conserved hydrophobic residues involved in forming the TMD-binding groove are labeled above with blue dots [86]. **B.** Phylogenetic reconstruction of the putative Get3 homologs in plasmodial lineage and with those corresponding to the yeast (*S*. *cerevisiae* and *S*. *pombe*), related apicomplexan (*T*. *gondii*) and kinetoplastid parasites (*L*. *donovani* and *T*. *cruzi*), plant (*A*. *thaliana*) and human (*H*. *sapiens)*. The evolutionary history was inferred by using the Maximum Likelihood method [138] and branch annotation was done using bootstrapping. The percentage of replicate trees in which the associated taxa clustered together in the bootstrap test is shown next to the branches [129] and evolutionary analyses were conducted in MEGA X [130]. **C.** Alignment of Phyre2 (*www.sbg.bio.ic.ac.uk/phyre2*) predicted 3D structure of PfGet3 (rainbow colored) with the crystal structure of ScGet3 (PDB ID 2WOJ; grey; [84]). The α- helices and β-sheets are numbered, and the core ATPase domain and α-helical subdomains are labeled. The positions of Mg^2+^ is shown (pink sphere). The P- and A-loops are indicated by arrows.

In eukaryotes, the conserved GET/TRC pathway has been implicated in targeting TA proteins through a concerted relay of events. Therein, the cytosolic ATPase Get3/TRC40 is located at the junctional interface and communicates with the pool of soluble components such as Sgt2/SGTA (hereafter referred to as Sgt2/A to incorporate features of both *H*. *sapiens* SGTA and *S*. *cerevisiae* Sgt2, as designated in [74]), Get4/5 or Bag6 at the upstream, and the downstream ER membrane bound receptors Get1/2 (or WRB/CAML) [75–77]. We thus focussed our efforts towards identifying the corresponding homolog of Get3 in *P*. *falciparum* and subsequently use it as a handle to reveal the identities of the homologous effectors of the GET machinery and the bound TA substrates in this parasite. Although there are >2,000 putative Get3s reported in KEGG and OrthoDB databases [78], no Get3 homolog has yet been reported in the malaria parasite *P*. *falciparum*. Thus, we retrieved the amino acid sequences of the Get3 from yeasts (*S*. *cerevisiae*; Uniprot ID Q12154, ScGet3; *Schizosaccharomyces pombe*, Uniprot ID Q9P7F8; SpGet3), plant (*A*. *thaliana*; Uniprot ID Q949M9; AtGet3a) and mammalian (*H*. *sapiens*; Uniprot ID O43681; HsGet3) systems from Uniprot (*www*.*uniprot*.*org*) and queried against the *P*. *falciparum* 3D7 database by BLAST in PlasmoDB. Results retrieved PF3D7_0415000 as the top hit in all queries. PF3D7_0415000 is a 379 amino acid protein annotated as a putative arsenical-pump driving ATPase, owing to its apparent ~17% identity and 32% similarity to the bacterial arsenite transporter ArsA which provides resistance to arsenite [79, 80] (**Figs 2A** and **S1**). Within eukaryotes, PF3D7_0415000 shared 46.9% similarity and 35.9% identity with ScGet3; 54.4% similarity and 41.8% identity with SpGet3; 51% similarity and 34.1% identity with AtGet3a and 59.7% similarity and 40.8% identity with HsGet3, respectively (**S1A** and **S1B Fig**). Like other Get3 members, PF3D7_0415000 also exhibited canonical features of the SIMIBI group of P-loop NTPase superfamily [81], including the presence of (i) a nucleotide hydrolase domain (NHD) containing a A-type motif called as Walker B with a conserved ‘P-loop’ (GGKGGVGKTT) that directly interacts with the phosphate moiety of the NTP substrates, (ii) a conserved motif B, composed of a hydrophobic β-strand and terminating in an Asp residue (SVIVFD) which is involved in interaction with Mg^2+^ for catalysis, (iii) the A-loop for adenosine recognition (QLKNEIR), and (iv) Switch I (DPAHN) and Switch II (DTAPTGHT) loops reported to undergo conformational rearrangements in the presence of γ-phosphate of ATP to form a ‘loaded spring’ that uncoils upon ATP hydrolysis [80, 82, 83]. However, the ‘CxxC motif’ characteristic of the eukaryotic Get3s (ScGet3, SpGet3 and HsGet3/TRC40) and implicated in the coordination of zinc ion for homodimerization was found to be absent in PF3D7_0415000 [84] (**Fig 2A**). Instead PF3D7_0415000 alignment displayed LxxC residues in which the first cysteine was replaced with leucine. Nonetheless, PF3D7_0415000 possessed a ~20 residue insertion called the Get3 motif or TRC40 insert that is deemed essential for the substrate TA binding. Thus, these conserved features prompted us to confidently annotate PF3D7_0415000 as PfGet3 throughout the course of this study. PfGet3 also showed a high level of conservation across the other members of *Plasmodium* species, including those that infect humans (*P*. *vivax* and *P*. *knowlesi*), as well as the other rodent-specific malaria parasites (*P*. *yoelii*, *P*. *chabaudi* and *P*. *berghei*) (**Fig 2B**). Phylogenetic analyses suggested that Get3 orthologs in the rodent malaria parasites diverge early in evolution and form a clade separate from the human counterparts. Get3 homologs has also been identified in other apicomplexan parasites, including *T*. *gondii* (TGVEG_231190), *T*. *cruzi* (Tc00.1047053507763.30), *L*. *donovani* (LDBPK_110710) and *C*. *parvum* (CGD7_4070) and they share varied similarity and identity to PfGet3 (**S1B Fig**).

Since there is no reported crystal structure of PfGet3, we submitted the amino acid sequence of PfGet3 to the Phyre2 server (*www.sbg.bio.ic.ac.uk/phyre2*; [85]) for *in silico* prediction of its secondary structure and disordered region. Phyre2 output revealed the presence of 51% α-helices, 10% β-strands and 21% disordered regions (**S2A Fig**). Submission of PfGet3 sequence to the TMHMM server (*www.cbs.dtu.dk/services/TMHMM-2.0*) predicted no transmembrane helix in PfGet3 consistent with PfGet3 being a soluble cytosolic protein (**S2B Fig**). The Phyre2 server also generated a 3D atomic model of PfGet3 based on remote homology and template-based assembly simulations to the existing Get3 crystal structures in the RCSB Protein Data Bank (PDB**; Fig 2C**). Although the predicted PfGet3 structure revealed 100% confidence with the Get3 orthologs from both prokaryotic and eukaryotic origins, it showed highest identity (59%) to the Get3 from the yeast *Debaryomyces hansenii* (DhGet3; PDB ID: 3IO3; 1.8 Å resolution) followed by 51% identity to both nucleotide-free SpGet3 (PDB ID: 2WOO; 3.01 Å resolution) and ADP-AlF_4_ complexed ScGet3 (PDB ID: 2WOJ, resolved at 2.3 Å), respectively [84, 86]. We preferred the model based on homology to the ScGet3 as the most-suitable predicted structure for PfGet3 because: (i) the ADP-AlF_4_-ScGet3/2WOJ has 99.3% sequence coverage compared to 91.4% for DhGet3/3IO3 (the finger domain of DhGet3 was missing in the electron density map); and more importantly, (ii) the crystal structure of multi-subunit GET translocation complex (Get3-Get4-Get5) from *S*. *cerevisiae* was also available in the PDB at 6 Å resolution (PDB ID: 5BWK) [87], which we believe would aid in further characterizing the GET components and their interactions in *P*. *falciparum* in future studies.

### Functional complementation in *S*. *cerevisiae Δget3* knockout strains and subcellular distribution of PfGet3 in *P*. *falciparum-*infected erythrocytes

**Fig 3:**
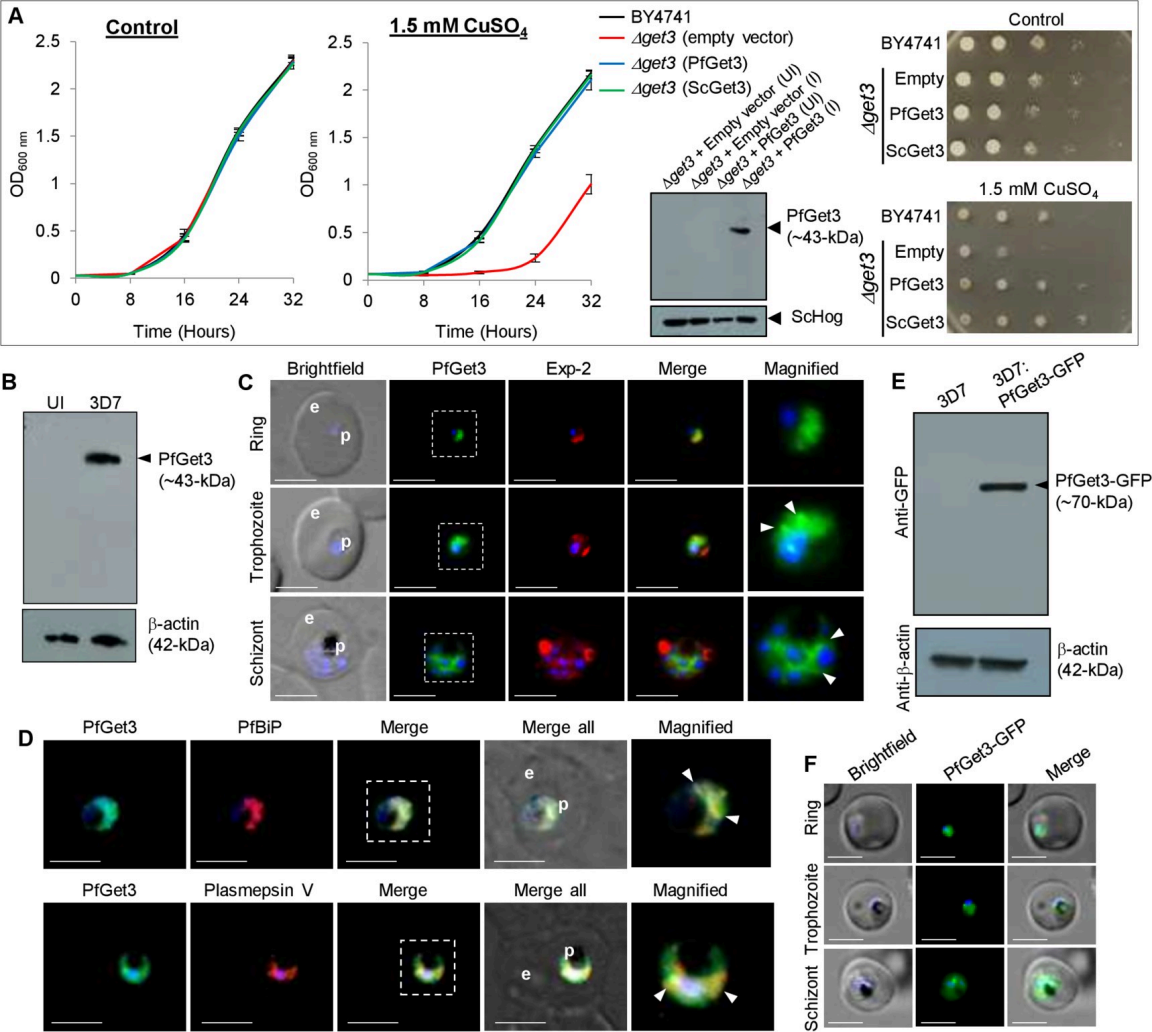
Functional complementation in the yeast *Δget3* knockout strain and localization of PfGet3 in *P*. *falciparum*-infected erythrocytes. **A.** Growth curves of *S*. *cerevisiae* BY4741 wild-type (black) or *Δget3* cells transformed with either empty vector (red), or with sequences encoding for PfGet3 (blue) or ScGet3 (green) at 30°C in the absence (left) or presence (right) of 1.5 mM CuSO_4_. While no growth differences were apparent in the absence of CuSO_4_, the salt-sensitive growth-defect in *Δget3* cells transformed with the empty vector was rescued by the expression of PfGet3 or ScGet3. Western blot confirms the expression of PfGet3 (arrowhead) in transformed *Δget3* cells only under induced condition (I) as compared to the uninduced (UI) control, using anti-PfGet3 antibodies (top). Anti-Hog antibodies detect *S*. *cerevisiae* Hog as a loading control (bottom). Corresponding images of the sequentially diluted plate-based growth assay in the absence (top) or presence of 1.5 mM CuSO_4_ (bottom) are shown at the extreme right. **B.** Western blot showing the expression of PfGet3 (arrowhead, top) at ~43-kDa in infected erythrocytes (3D7; right lane) but not in uninfected (UI; left lane) red blood cells. Antibodies to the human β-actin (42-kDa) was used as a loading control (bottom). **C.** IFA images showing the expression and localization of PfGet3 (green) as diffused cytosolic fluorescence in the ring (top panel), trophozoite (middle panel) and schizont (bottom panel) stages of infection. In trophozoites and schizonts, additional punctate spots or reticular staining of PfGet3 structures (arrowheads) can be seen in the magnified versions of the dotted outlined regions. The parasite boundary is marked by antibodies to the parasitophorous membrane protein, PfExp-2 (red). **D.** IFA images showing areas of colocalization between PfGet3 (green) and the ER-resident protein markers such as BiP (red; top panel) or Plasmepsin V (red; bottom panel), as indicated by arrowheads in the magnified view of the dotted outlined regions. **E.** Western blot showing the expression of PfGet3-GFP fusion protein (arrowhead; top) at ~70-kDa in transgenic parasites (3D7: PfGet3-GFP, right lane) but not in the parental 3D7 (left lane). Antibodies to the human β-actin (42-kDa) was used as a loading control (bottom). **F.** Live cell images showing the distribution of PfGet3-GFP (green) in transgenic parasites. For all IFA images, corresponding brightfield, single or merged fluorescent images with nuclear staining (Hoechst 33342; blue) are as shown. For all live cell, brightfield and IFA images are shown; p and e denote parasite and erythrocyte, respectively. Scale bar = 5 μm.

A null mutant in *S*. *cerevisiae* Get3 (*Δget3*) has been reported to exhibit no phenotypic growth defects in synthetic complete media (or rich media), or changes in the cellular morphology, colony size, mating, sporulation, or germination [88]. Instead, *S*. *cerevisiae Δget3* strains are found to be more sensitive to As^3+^-, As^5+^-, Co^2+^-, Cr^3+^- and Cu^2+^-induced stress and high temperature-mediated stress than the parental wild-type strain [89]. To investigate the comparable function between PfGet3 and ScGet3, we transformed *S*. *cerevisiae Δget3* strain with either empty vector or those containing sequences encoding for PfGet3 or ScGet3 and assessed their growth in the absence or presence of 1.5 mM CuSO_4_. The expression of PfGet3 at ~43-kDa in transformed *Δget3* strain was confirmed by western blotting using custom-generated antibodies against PfGet3 (**Fig 3A**). Growth kinetics experiments revealed no apparent differences in growth between the wild-type BY4741 and *Δget3* strains in the absence of CuSO_4_. However, the *Δget3* strain transformed with the empty vector displayed a salt-sensitive growth phenotype consistently seen both across the liquid culture-based and the plate-based growth assay. This phenotype was successfully rescued by the *in trans* expression of PfGet3 to a similar level as seen in the wild type BY4741 cells or in the transformed *Δget3* knockout strain expressing ScGet3 (**Fig 3A**). These results indicated that PfGet3 possessed characteristics like the ScGet3 in rescuing the salt-sensitive phenotype in *Δget3* knockouts.

Transcriptomic data in PlasmoDB indicates two waves of *pfget3* transcriptions in *P*. *falciparum* 3D7 (refer to *www.plasmodb.org* for the transcriptomic data): an initial high wave in ring stages within the first 10 hours post-invasion (hpi) and followed by a short shoulder at 20–36 hpi trophozoite stages, thus suggesting a requirement of PfGet3 function in the intraerythrocytic developmental cycle. To further investigate the expression of PfGet3 during the intraerythrocytic stages, the custom generated polyclonal anti-PfGet3 antibodies (see *Materials and Methods*) were validated in western blots using *P*. *falciparum* 3D7 parasites or with the purified recombinant PfGet3. Results indicated specific recognition at ~43-kDa corresponding to the predicted molecular weight of PfGet3 as 43.3-kDa in *P*. *falciparum* parasites but not in uninfected erythrocytes (**Fig 3B**). These anti-PfGet3 antibodies also recognized the recombinant 6×his tagged PfGet3 expressed in *E*. *coli* cells (**S3 Fig**).

Anti-PfGet3 antibodies were also used in indirect IFA to assess PfGet3 localization within 3D7 parasites. IFAs predominantly showed a diffused PfGet3 fluorescence within the confines of the parasite boundary (demarcated by antibodies to the parasitophorous vacuolar membrane protein Exp-2), thus indicative of the parasite cytosolic staining of PfGet3 in the ring stage parasites (**Fig 3C**, top panel). However, in trophozoites and schizonts additional punctate/reticulate PfGet3 fluorescence were also detected in the perinuclear region characteristic of the parasite ER compartments (**Fig 3C**, arrowheads in the middle and bottom panels). We were unable to visualize these concentrated PfGet3 patterns in the young rings owing to their small size and limitations in image resolution. In trophozoites and schizonts, the concentrated puncta of PfGet3 showed good colocalization with the ER chaperone PfBiP and the ER-resident parasite protease Plasmepsin V (**Fig 3D**) thereby suggesting that a fraction of PfGet3 was at the vicinity of the parasite ER. These observations were consistent with the previously reported localization profiles of ScGet3 and the mammalian TRC40 in the perinuclear/ER compartments [90, 91]. This further implied the existence of two different pools of PfGet3; a cytosolic soluble pool that possibly interacts with homologs other soluble cytosolic GET components (such as SGT2/A, Get4 and Get5/UBL4A) for accessing the TA substrates; and an ER-membrane associated pool wherein the TA-bound Get3 interacts with the homologs of receptors like Get1/WRB and Get2/CAML for mediating TA-insertion into the ER. It is pertinent to mention that no homologs of the other GET components or the existence of a functional GET pathway has been identified in *P*. *falciparum* to date. Similarly, we also generated transgenic parasites expressing PfGet3-GFP, and western blots confirmed the expression of a fusion protein at ~70-kDa (**Fig 3E**). Live cell microscopy of PfGet3-GFP transgenic parasites further showed the presence of the diffused cytosolic fluorescence as well as punctate PfGet3-GFP spots in the perinuclear region, thus confirming the distribution profiles seen in the IFA images (**Fig 3F**). Overall, the subcellular distribution of PfGet3 and its ability to reverse the CuSO_4_-sensitivity in the *Δget3* yeast knockout strain, encouraged us to further investigate and identify the other homologs of the GET machinery in this parasite.

### Proximity labelling BioID approach indicates association between PfGet3 and multiple TA-proteins

**Fig 4:**
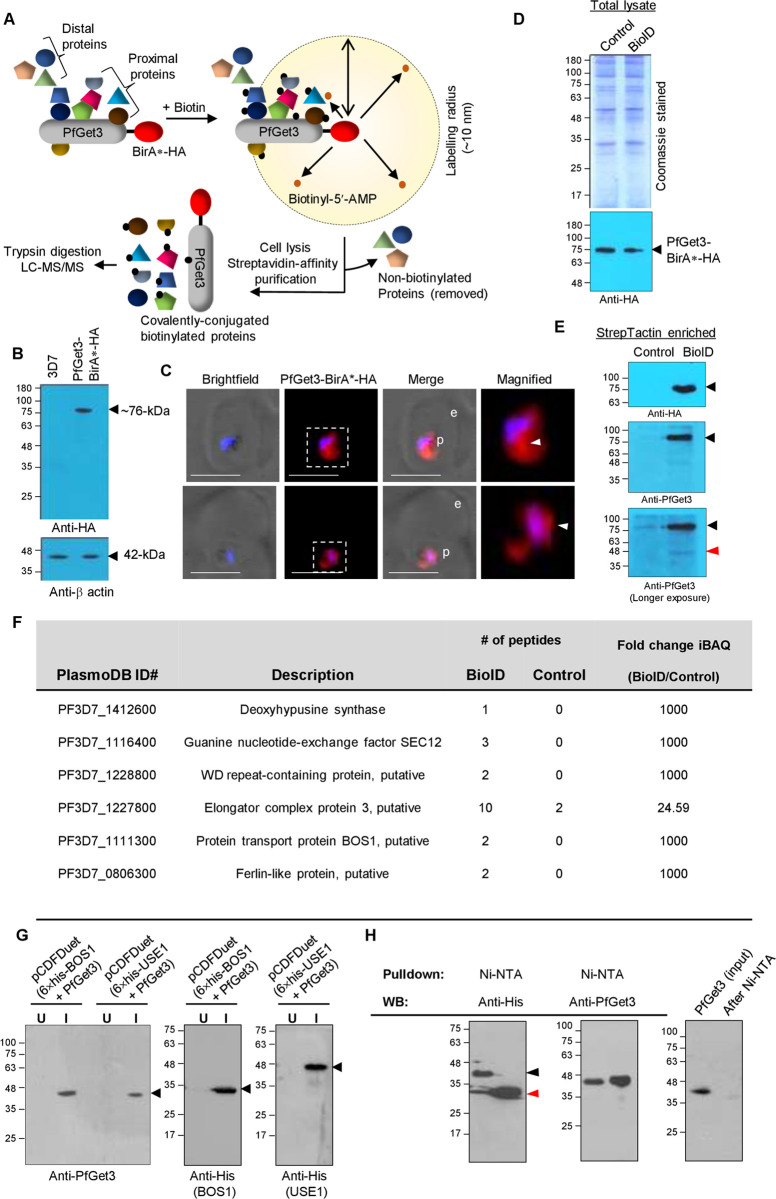
Proximity biotinylation using PfGet3-BirA*-HA and its association with the TA proteins. **A.** Schematic for proximity biotinylation by the bacterial biotin ligase BirA* (red) fused to the C-terminus of PfGet3 (with a HA tag downstream). BirA* converts exogenously added free biotin to highly reactive biotinyl-5-AMP (small orange dots) which reacts with the primary amines of proximal proteins (within 10 nm radius, interacting directly or indirectly) and cause their biotinylation (indicated by small solid black dots). Distal proteins beyond the 10 nm radius are not biotinylated irrespective of their interactions with PfGet3-BirA*-HA. Following biotinylation, the biotinylated proteins are affinity purified from cellular lysates using streptavidin beads and identified by LC-MS/MS [139]. **B.** Western blot showing the expression of ~76-kDa PfGet3-BirA*-HA (arrowhead, top) only in transgenic parasites (right lane) but not in the parental 3D7 parasites (left lane). Antibodies to β-actin at ~42-kDa (bottom) are used as loading control. **C.** IFA images showing the cytosolic distribution of PfGet3-BirA*-HA (red) in transgenic parasites. Boxed areas (dotted outlines) are magnified at the right to highlight areas of concentrated PfGet3-BirA*-HA (arrowheads). Corresponding brightfield and fluorescent images are as indicated. Parasite nucleus (blue) is stained with Hoechst 33342; scale bar = 5 μm; p, parasite; e, erythrocyte. **D.** Coomassie-stained SDS-PAGE (top) and western blot (bottom) of the total lysates from transgenic parasites expressing PfGet3-BirA*-HA grown in the absence (control) or the presence (BioID) of D-biotin. The Coomassie-stained gel indicates equal loading and the western blot using antibodies to the HA tag depicts similar PfGet3-BirA-HA expression under both conditions. **E.** Western blots showing the presence of PfGet3-BirA*-HA protein (~76-kDa; black arrowheads) only in the StrepTactin affinity-purified BioID fraction and not in the control, using antibodies to the HA-tag (top) or PfGet3 (middle and bottom). The bottom blot represents a longer exposure of the middle blot to highlight the detection of native PfGet3 (red arrowhead), possibly due to homomer formation with PfGet3-BirA*-HA. **F.** Table showing the 6 predicted TA proteins enriched in the BioID fraction as compared to the control. The corresponding PlasmoDB ID and descriptions are as mentioned along with the relative number of peptides detected by LC-MS/MS. Proteins not identified in the control samples were assigned iBAQ fold-change value of 1000 (also see *S1 Text* and *Table 2*). **G.** Western blots detecting the expression of recombinant proteins (arrowheads): PfGet3 (left), 6×his-PfBOS1 (middle) and 6×his-PfUSE1 (right) in transformed *E*. *coli* on induction with IPTG (I) as compared to the uninduced control (U). **H.** Western blots showing the co-association of recombinant PfGet3 (middle) with the Ni-NTA pulldown fractions of recombinant 6×his-PfBOS1 (red arrowhead; left) or 6×his-PfUSE1 (black arrowhead; left). The blot at the right highlights no non-specific association of recombinant PfGet3 to the Ni-NTA resin. Molecular weight standards for all the SDS-PAGE gels and blots (in kDa) are as indicated.

BioID harnesses the potential of biotin ligase BirA* (BirA with a R118G mutation) to promote promiscuous biotinylation. When fused to a ‘bait’ protein, the ligase catalyses a two-step reaction in the presence of biotin to generate reactive bioAMP that covalently biotinylates adjacent primary amines of the proximate ‘prey’ proteins [92]. These biotinylation events are then effectively enriched using streptavidin affinity matrices and permit the identification of dynamic or weak interactions largely lost during the complicated (immuno)-purification protocols (*see schematic in* ***Fig 4A***). Since the interactions of TA proteins with Get3 are deemed transient; to experimentally validate our bioinformatic predictions of TA proteins in *P*. *falciparum* 3D7 and their association with PfGet3, we generated transgenic parasites expressing PfGet3 fused to BirA*-HA at the C-terminus (see *Materials and Methods*). Expression was confirmed in western blots using antibodies to the HA tag, which detected PfGet3- BirA*-HA fusion protein of ~76-kDa (**Fig 4B**). IFA of the PfGet3-BirA*-HA-expressing parasites showed diffused distribution within the parasite cytosol (**Fig 4C**). We were unable to find any concentrated fluorescent puncta of PfGet3-BirA*-HA similar to those seen in the native PfGet3 images (**Fig 3C, 3D and 3F**), likely either due to the increased intensity of cytosolic PfGet3-BirA*-HA fluorescence or due to the use of antibodies to the HA-tag. However, in magnified images we noticed relative enrichment areas of PfGet3-BirA*-HA near the perinuclear region (arrowheads in **Fig 4C**). Equal volumes of total extracts from PfGet3-BirA*-HA cells grown either in the absence (control fraction) or in the presence of D-Biotin (named as the BioID fraction hereafter) (**Fig 4D**) were then used to affinity purify the proximal biotinylated interactors using StrepTactin XT beads and identified either by western blotting or LC-MS/MS. In the western blots using antibodies to both HA tag and PfGet3, the PfGet3-BirA*-HA was enriched only in the BioID fraction (**Fig 4E**) although it could be detected in the crude lysates of both the control and BioID fractions (**Fig 4D**). Interestingly, antibodies to PfGet3 also detected the native untagged PfGet3 on longer exposure of the membrane thereby implying the formation of a ‘heteromer’ (possibly dimer, tetramer, or higher oligomerization states) between the untagged PfGet3 and PfGet3-BirA*-HA and thus the ensuing biotinylation. Self-biotinylated PfGet3-BirA*-HA was also detected in western blots. Mass spectrometry results yielded a list of proteins enriched in the BioID fraction of PfGet3-BirA*-HA as compared to the control sample (*see* ***S2 Table*** *for the complete list*). PfGet3 was one of the top hits in the BioID fraction with 24 different peptides and ~10-fold excess over the control. Since the other homologs of Get3 are confirmed to form oligomers, we speculate that this low fold change might have been influenced either by the sample size, the overall abundance of PfGet3 (even when the transgenic parasites express PfGet3-BirA*-HA under the constitutive *cam* promoter), the ability of PfGet3-BirA*-HA to self-biotinylate or due to the low resolution LC separation from complex protein mixtures (*e*.*g*., crude cell lysate) [93]. The identified peptides from PfGet3-BirA*-HA may have been sourced either from self-biotinylated PfGet3-BirA*-HA or *trans*-biotinylated native PfGet3 (**Fig 4E**). These also included two biotinylated peptides GTLNVL**K**NFTNNEMEFDSLYEK and **K**EMFDNILPELLHSFPGIDEALCFAELMQSIK (**K** indicates the site of biotinylation), thus confirming successful recruitment of the biotinylizer.

Peptides corresponding to six predicted TA proteins were also quantitatively enriched in the BioID fraction (**Fig 4F**). These included PF3D7_0806300 (Ferlin-like protein, putative), PF3D7_1111300 (Protein transport protein BOS1, putative), PF3D7_1116400 (Guanine nucleotide-exchange factor SEC12), PF3D7_1227800 (Elongator complex protein 3, putative), PF3D7_1228800 (WD repeat-containing protein, putative) and PF3D7_1412600 (Deoxyhypusine synthase), and accounted for approximately 43% and 10% of the 14 ER-predicted and the total 63 predicted TA proteome, respectively in *P*. *falciparum*. We also observed that the sum of peptides derived from each TA protein was limiting and we were unable to find biotinylation in any of the peptides corresponding to these proteins. This could either be due to the sample size or due to their low abundance or transient interactions with PfGet3-BirA*-HA that limits the scope of biotinylation at all the possible sites within these TA proteins. Nonetheless, we strongly believe that these TA proteins were biotinylated since they were affinity-purified with the StrepTactin-resin and enriched in the BioID fraction as compared to the control. Among the enriched TA proteins, PF3D7_1111300 and PF3D7_1116400 have been predicted in the ER-localized TA family in this study and corresponding homologs already validated in other systems [94, 95]. Although the remaining hits; PF3D7_0806300, PF3D7_1227800, PF3D7_1228800 and PF3D7_1412600 belong to TA proteins localized to other destinations (**S1 Table**), it is possible that their targeting in *P*. *falciparum* intersects with the GET pathway involving PfGet3. Interestingly, our BioID fraction was devoid of any mitochondrial TA proteins, an expected result since mitochondrial TA targeting essentially recruits GET/TRC-independent mechanisms [11, 96–98]. Similarly, even within the subset of ER-resident TA proteins, other trafficking mechanisms are known to exist that preferentially utilize the SRP or the Hsc70-Hsp40 systems instead of the GET/TRC route [21, 99–101], thus explaining the low numbers of TA proteins detected in our BioID fraction. A recent study also revealed the inability of TRC40 (the mammalian homolog of PfGet3) to effectively engage TA proteins with moderately hydrophobic TMD. These proteins are instead shielded off from the hydrophilic cytosol by calmodulin (and not by Sgt2/A) and inserted at the ER *via* the conserved ER membrane protein complex (EMC) rather than the GET pathway [25]. Within the ER-specific TAs grouped in this study, we found at least six proteins (PF3D7_0323100, PF3D7_0618700, PF3D7_0627300, PF3D7_0821800, PF3D7_0826600 and PF3D7_1032400) exhibit comparatively lower GRAVY scores (<2.0) with respect to the others (with GRAVY scores >2.0), which might have contributed towards the absence of their biotinylation and ‘capture’ in our PfGet3 BioID fraction. Taking this into account, our BioID fraction essentially detected 2/8 (or 25%) of the TA proteins that were most plausible to recruit the GET pathway for ER trafficking. However, the precise GRAVY score that decides preference for the GET (in)dependent pathway is currently unknown in *P*. *falciparum* and further experiments are warranted to ascertain their detailed characterization. To further validate the binding of TA proteins with PfGet3, we selected two representative TAs for our co-association studies. While one of the candidates, *i*.*e*., PF3D7_1111300 (PfBOS1) was enriched in our BioID fraction (**Fig 4F**), we did not detect any peptides corresponding to PF3D7_0710800 (PfUSE1) by LC-MS/MS. Pulldown from the soluble crude lysates of transformed *E*. *coli* co-expressing recombinant 6×his-PfBOS1 or 6×his-PfUSE1 along with the recombinant untagged PfGet3 (**Fig 4G**; left and middle blot) using Ni^2+^-NTA resin and detection by western blotting identified PfGet3 in the Ni^2+^-NTA purified 6×his-PfBOS1 or 6×his-PfUSE1 fractions, thereby confirming a direct binding of PfGet3 to these TA proteins. The recombinant PfGet3 (when singly expressed in the same plasmid vector) did not display any Ni^2+^-NTA-binding characteristic (**Fig 4G**; right blot). These co-association studies further suggested that multiple TA proteins likely interacted with PfGet3 in the proximity biotinylation assay but were not detected in LC-MS/MS due to a paucity in their peptide abundance, limited sample size or the detector sensitivity.

### PfGet3 also interacts with a putative homolog of Get4 in *P*. *falciparum*

**Fig 5:**
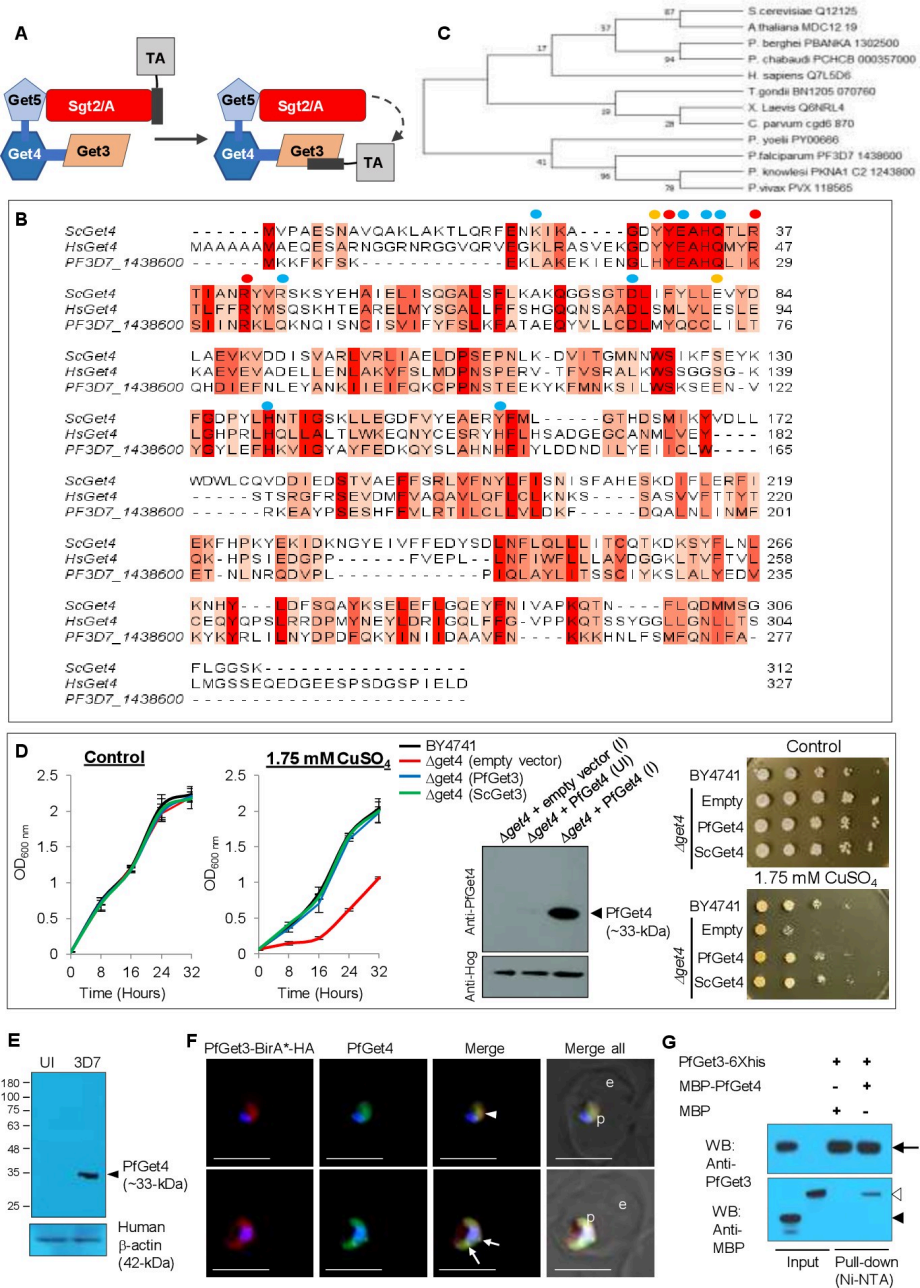
Identification of PfGet4 and its association with PfGet3. **A.** Schematic showing the interactions between Get3, Get4, Get5 and Sgt2/A. The Sgt2/A-associated TA protein is subsequently transferred to Get3 (dotted arrow). **B.** Sequence alignments of the three eukaryotic Get4 homologs (PF3D7_1438600/PfGet4, ScGet4 and HsGet4) highlighted in the different shades of red based on the extent of conservation. Residues involved in Get4-Get3 interface contacts are indicated by spheres above and colored by the phenotype (blue, none or minimal; orange, moderate; red, strong; as according to [140]). **C.** Phylogenetic reconstruction of Get4 proteins in plasmodial lineage with the homologs from yeast (*S*. *cerevisiae*), related apicomplexans (*T*. *gondii* and *C*. *parvum*), *X*. *laevis*, plant (*A*. *thaliana*) and human (*H*. *sapiens)*. The evolutionary history was inferred by using the Maximum Likelihood method [138] and branch annotation was done using bootstrapping. The percentage of replicate trees in which the associated taxa clustered together in the bootstrap test are shown next to the branches [129] and evolutionary analyses were conducted in MEGA X [130]. **D.** Growth curve of *S*. *cerevisiae* BY4741 wild-type (black) or *Δget4* cells transformed with either the empty plasmid (red) or with sequences encoding for PfGet4 (blue) or ScGet4 (green) grown at 30°C in the absence (left) or the presence of 1.75 mM CuSO_4_ (right). While no growth differences are apparent in the absence of CuSO_4_, the salt-sensitive growth defect exhibited by *Δget4* cells transformed with the empty plasmid (red) is rescued by the expression of PfGet4 or ScGet4 (green). Western blot at the right confirms the expression of PfGet4 (arrowhead) in transformed *Δget4* cells only under induced condition (I) as compared to uninduced (UI) control, using custom generated anti-PfGet4 antibodies (top). Anti-Hog antibodies detect *S*. *cerevisiae* Hog as the loading control (bottom). Corresponding images of the plate-based growth assay in the absence (top) or presence of 1.75 mM CuSO_4_ (bottom) are shown at the extreme right. **E.** Western blot showing the expression of PfGet4 (arrowhead, top) at ~33-kDa in infected erythrocytes (3D7, right lane) but not in uninfected (UI, left lane) red blood cells. Antibodies to the human β-actin (42-kDa) were used as loading control (bottom). **F.** IFA images of ring (top panel) and trophozoite (bottom panel) showing the areas of colocalization (yellow) between PfGet3-BirA*-HA (red) and PfGet4 (green) indicated by an arrowhead (in ring; top) or arrows (in trophozoite; bottom) in the merged view of fluorescence. Corresponding brightfield, single or merged fluorescence images with nuclear staining (Hoechst 33342; blue) are shown. Scale bar = 5 μm; e, erythrocyte; p, parasite. **G.** Western blots showing the association of purified 6×his tagged recombinant PfGet3 (~43-kDa; black arrow) with MBP-PfGet4 (empty arrowhead) but not with MBP (empty position indicated by a solid arrowhead) as revealed by the pull-down assay using Ni^2+^-NTA beads followed by western blots using antibodies to MBP (bottom) or PfGet3 (top). Corresponding input and pull-down lanes are as indicated. Molecular weight standards (in kDa) are as shown for all the SDS-PAGE and western blot images.

Our BioID fraction also enriched 8 peptides from PF3D7_1438600, while only one was detected in the control (***S2* Table**). Interestingly, PF3D7_1438600 was also the only significant hit in BLAST searches with the amino acid sequences of the well-characterized Yor164c (the *S*. *cerevisiae* homolog of Get4) and the *H*. *sapiens* Golgi to ER traffic protein 4 homolog/TRC35 (HsGet4) against *P*. *falciparum* 3D7 proteome, with significant e-values of 2e-4 and 9e-11, respectively. PF3D7_1438600 encodes for a 277 amino acid cytosolic protein, without any ER-type secretory signal sequence or TMD and has been annotated as a conserved protein with a domain of unknown function (DUF410) in PlasmoDB. PF3D7_1438600 shares considerable similarity with Yor164c (17.6% identity and 36.3% similarity), HsGet4/TRC35 (15.6% identity and 37.8% similarity) as well as other Get4 homologs (**Figs 5B** and **S4A–S4C**), including the residues involved in interactions with Get3 [102, 103]. Homologs of Get4 form a hetero-tetrameric complex with Get5 and mediates efficient delivery of TA substrates from Sgt2 to Get3 (*schematic in* ***Fig 5A****; [34]*). Evolutionary analyses of the orthologs of Get4 by the Maximum-likelihood method [104] revealed PF3D7_1438600 in a clade with other *Plasmodium* parasite species that infect humans (**Fig 5C**). Surprisingly, the Get4 ortholog of the rodent malaria parasite *P*. *yoelii* (PY00666) also grouped closer to the human malaria parasites (*P*. *falciparum* and *P*. *knowlesi*) than other rodent malaria species (*P*. *berghei* and *P*. *chabaudi*). We thus annotated PF3D7_1438600 as PfGet4 hereafter in this study. Prior structural studies have shown that yeast Get4 is an alpha-helical repeat protein with the N-terminus (residues 1–148) involved in Get3 binding and its C-terminus interacting with Get5 [102, 103]. Since no structural information about PfGet4 is available, we submitted PfGet4 amino acid sequence to the Phyre2 server (*www.sbg.bio.ic.ac.uk/phyre2*) for *in silico* secondary structure prediction. Phyre2 output revealed the presence of 72% α-helices and 14% disordered regions (**S4B Fig**). PfGet4 structure showed 100% confidence with the crystal structure of the S. *cerevisiae* Get4-Get5 complex (PDB ID: 2WPV) and the human Bag6-NLS & TRC35 complex (PDB ID: 6AU8; **S4D Fig**). Essentially, PfGet4 was also predicted to form 14 right-handed helices arranged pairwise with an α-solenoid like ScGet4 [102, 103]. *In trans* expression of PfGet4 (and confirmed by western blotting) also rescued the CuSO_4_-sensitive growth phenotype in *Δget4* strain (as compared to those transformed with the vector only) to a similar level as the wild-type BY4741 cells or *Δget4* cells transformed with ScGet4 in both the liquid culture-based or the plate-based growth assays (**Fig 5D**). Custom anti-peptide antibodies against PfGet4 (as described in the *Materials and Methods*) detected a parasite protein at ~33-kDa (predicted molecular weight 33,150-Da) in infected erythrocytes but not in uninfected RBC (**Fig 5E**). IFA revealed localization of PfGet4 as diffused fluorescence indicative of the parasite cytosolic staining. Immunolocalization studies in transgenic parasites expressing PfGet3-BirA*-HA using mouse-anti-HA antibodies in conjunction with anti-PfGet4 (both our anti-PfGet3 and anti-PfGet4 antibodies were generated in rabbits, so we were unable to use them in parallel) showed diffused cytosolic staining pattern for PfGet4 with some areas of colocalization (arrowhead showing the yellow overlap region in the top panel of **Fig 5F**). In some late stage trophozoites, we also observed a few punctate spots of PfGet4 fluorescence in the parasite cytosol that were devoid of PfGet3 (arrows in the bottom panel of **Fig 5F**). These likely represent the pre-targeting PfGet4 complex (PfGet4-PfGet5-PfSgt2/A-TA) prior to its association with PfGet3. To assess the interaction between PfGet4 and PfGet3 *in vitro*, we expressed and purified recombinant MBP-PfGet4 in *E*. *coli* (see *Materials and Methods*). Purified recombinant MBP-PfGet4 or recombinant MBP (as a control) were incubated with recombinant PfGet3-6×his and affinity-purified using Ni^2+^-NTA beads to assess co-association. Western blots using anti-MBP antibodies confirmed the selective binding of MBP-PfGet4 (but not MBP) to PfGet3 (**Fig 5G**). Based on these observations and our earlier BioID results, we reasonably concluded that the *P*. *falciparum* genome encodes for a functional PfGet4 with features similar to the other Get4 homologs, and PfGet4 directly interacts with PfGet3, as expected for the canonical GET pathway.

### Other predicted homologs of *P*. *falciparum* GET machinery are also enriched in the BioID fraction

Get3 is a central component of the GET pathway, orchestrating connections between the TA-recognition complex (as it captures the TA substrates from the emerging ribosomal tunnel) and the downstream Get1/2 receptors at the ER membrane that functions to insert the TA proteins. Thus, the transgenic PfGet3-BirA*-HA parasites also offered possibilities to biotinylate both upstream and downstream effectors of the GET pathway. We therefore mined the BioID fraction further to identify the other GET machinery homologs in *P*. *falciparum*. The TRC comprises of the yeast cochaperone Sgt2 (or SGTA; the equivalent mammalian homolog) and the scaffolding proteins Get4 (or TRC35, the mammalian homolog) and Get5 (or mammalian Bag6 complex) [11]. The binding of the TA proteins to Sgt2/A is considered as the first committed step for the GET/TRC trafficking pathway (**Fig 5A**; [34, 77, 105]). However, the role of cytosolic chaperones like the yeast cytosolic Hsp70, Ssa1 and Hsp40 in harnessing the energy of ATP hydrolysis to unfold aggregated TA substrates and preserve their conformational quality prior to Sgt2/A loading has also been reported [33]. The central region of Sgt2/A contains the conserved TPR domains that associates with additional heat-shock proteins like Hsp70, Hsp90 and Hsp104 [34, 106, 107]. Heat-shock cognate 70 (Hsc70) has been found to be associated with *in vitro* translated TA proteins from mammalian lysates and mutations in the residues of the TPR domain which prevent Hsp70 binding impairs the loading of TA substrates onto Sgt2/A [21, 33]. We observed a selective enrichment of heat-shock proteins like PfHsp90 (PF3D7_0708400), multiple PfHsp70s (PF3D7_0818900, PF3D7_0917900/BiP, PF3D7_1134000), PfHsp110 (PF3D7_0708800), PfHsp60 (PF3D7_1015600), PfHsp101 (PF3D7_1116800) and small heat shock protein (PF3D7_1304500) in our BioID fraction (**Tables 2** and ***S2***). Neither PfGet3 nor PfGet3-BirA*-HA display any obvious chaperone-binding motif, so we speculated that the proximal presence of the plasmodial homologs of Sgt2/A (PfSgt2/A) with Hsp-recruitment potential to PfGet3-BirA*^-^HA ensured their biotinylation and enrichment in the BioID fraction. To identify the putative PfSgt2/A, we queried *P*. *falciparum* 3D7 proteome with amino acid sequences of ScSgt2 or HsSGTA. Results retrieved multiple hits with significant e-values, such as PF3D7_1434300 (Hsp70/Hsp90 organizing protein/PfHOP), PF3D7_1241900 (Tetratricopeptide repeat protein), PF3D7_0213500 (Tetratricopeptide-repeat protein), PF3D7_1355500 (Serine threonine protein phosphatase 5), PF3D7_0707700 (E3 ubiquitin ligase) and PF3D7_1334200 (Chaperone binding protein), respectively. Unfortunately, our LC-MS/MS analyses did not yield any peptide corresponding to PfHOP, however, we detected of 12, 4, 3 and 2 peptides corresponding to PF3D7_0707700, PF3D7_1334200, PF3D7_1355500 and PF3D7_1241900, respectively in the BioID fraction and none were detected in the control (***S2* Table**). Interestingly, all these proteins contain TPR domains and could likely recruit the Hs(c/p) cascade to function as a putative PfSgt2/A. Indeed, a subclass of TPR-domain containing proteins such as Protein phosphatase 5, HOP and Sti1 in yeast act as cochaperones in regulating the nucleotide hydrolysis cycles and connecting protein folding pathways with the alternate route to ubiquitination and degradation [108]. However, the involvement of PfHOP also cannot be ruled out solely because no peptides were detected in the ‘nonsaturable’ LC-MS/MS and further experiments are required to identify and validate a cognate PfSgt2/A in *P*. *falciparum*.

**Table 2:** List of the confirmed or shortlisted homologs of the GET pathways in *P*. *falciparum* 3D7 identified in this study. Proteins are listed according to their PlasmoDB ID and descriptions. Rows showing the validated homologs of the GET machinery in *P*. *falciparum* are in bold and highlighted in grey, while shortlisted putative homologs without confirmed identities are in regular text. The fold change for each protein is measured as the ratio of iBAQ values between the BioID and control fractions (*see **S2 Table** for more details*).

Component of the GET pathway	Putative *P*. *falciparum* homolog	Description in PlasmoDB	Peptides in BioID	Fold change iBAQ (BioID/Control)_†_	Status (this study)
Sgt2/A	PF3D7_1434300	Hsp70/Hsp90 organizing protein HOP	No	NA	Shortlisted
	PF3D7_1241900	Tetratricopeptide repeat protein	Yes	1000	Shortlisted
	PF3D7_0213500	Tetratricopeptide-repeat protein	No	NA	Shortlisted
	PF3D7_1355500	Serine threonine protein phosphatase 5	Yes	1000	Shortlisted
	PF3D7_0707700	E3 ubiquitin ligase	Yes	1000	Shortlisted
	PF3D7_1334200	Chaperone binding protein	Yes	1000	Shortlisted
**Get4/TRC35**	**PF3D7_1438600 (PfGet4)**	**Conserved protein, unknown function**	**Yes**	**1000**	**Confirmed**
Bag6/UBL4A	PF3D7_0922100	Ubiquitin-like protein	Yes	1000	Shortlisted
	PF3D7_1211800	Polyubiquitin	No	NA	Shortlisted
	PF3D7_1313000	Ubiquitin-like protein Nedd8 homolog	No	NA	Shortlisted
Get5	PF3D7_1313000	Ubiquitin-like protein Nedd8 homolog	No	NA	Shortlisted
**Get3**	**PF3D7_0415000 (PfGet3)**	**Arsenical pump-driving ATPase**	**Yes**	**9.26**	**Confirmed**
**Get2/CAML**	**PF3D7_0215600**	**Conserved Plasmodium protein, unknown function**	**Yes**	**1000**	**Confirmed**
Get1/WRB	ND	ND	ND	NA	Unidentified

† Proteins not identified in the control samples were assigned iBAQ fold-change value of 1000.

The mammalian Bag6 complex also functions as an upstream loading factor, delivering TA substrates to the TRC40 (the mammalian ortholog of Get3) [19, 109]. Comprising of Bag6 (BCL-2-associated athanogene cochaperone 6; also known as BAT3 or *Scythe*), TRC35 (HsGet4) and Ubl4A (Ubiquitin-like protein 4A), the Bag6 complex associates with the ribosomes and awaits the emergence of TMDs from the ribosomal tunnel [35, 110]. The translating TA proteins interact with HsSGTA via Bag6, thus triaging HsSGTA function to ER-associated degradation (ERAD) and proteasomal activity [64, 111, 112]. We thus envisioned that the mammalian Bag6 complex in *P*. *falciparum* could possibly be biotinylated during its proximity to the putative PfSgt2/A as it ‘transfers’ the TA substrates to PfGet3-BirA*-HA, and accordingly get affinity-purified. PF3D7_0922100 was the top hit for *H*. *sapiens* Bag6 BLAST search against *P*. *falciparum* in PlasmoDB with an e-value of 2e-08. However, other hits from the BLAST query, like PF3D7_1113400, PF3D7_1365900, PF3D7_1313000 and PF3D7_1211800 also exhibited significant e-values of 1e-05, 3e-06, 2e-05 and 3e-04, respectively. Our BioID fraction detected 14 peptides corresponding to PF3D7_0922100, while none was detected in the control fraction (**Tables 2 and *S2***). Surprisingly, BLAST query with the amino acid sequence of the human Ubl4A also yielded plasmodial proteins overlapping with the Bag6 hits, notably PF3D7_1211800, PF3D7_1365900, PF3D7_0922100, PF3D7_1113400 and PF3D7_1313000 with e-values of 8e-12, 3e-12, 3e-08, 1e-07 and 2e-08, respectively. PF3D7_1365900 is 128 amino acids in length and annotated as ubiquitin-60S ribosomal protein L40. It was discarded as a candidate PfBag6/PfUbl4A because *firstly*, peptides were detected both in the BioID and control fraction (with only 5.03-fold enrichment), and *secondly* because PF3D7_1365900 has been solved as a part of the *P*. *falciparum* 80S ribosome by cryo-EM (PDB ID: 3J79; [113]. Similarly, PF3D7_1113400, annotated as a putative ubiquitin domain-containing protein DSK2, was also discarded since the crystal structure of the UBL domain of PF3D7_1113400/PfDsk2 (PDB ID: 6JL3) looks remarkably similar to the crystal structure of the complex between the UBA and UBL domains of *S*. *cerevisiae* Dsk2 (ScDsk2; PDB ID: 2BWE) [114, 115] and no role of ScDsk2 in the well-characterized GET pathway of yeast has been demonstrated. We, however, retained the other hits including PF3D7_1211800, a 381 amino acids protein designated as Polyubiquitin (PfpUB); PF3D7_0922100, a 1542 amino acids protein annotated as a putative ubiquitin-like protein; and PF3D7_1313000, a 76 amino acids protein annotated as a putative Ubiquitin-like protein Nedd8 homolog, as potential candidates for Bag6/Ubl4A in *P*. *falciparum* since they exhibit conservation in the critical residues implicated in the binding of Bag6-UBL to the CUE (coupling of ubiquitin conjugation to endoplasmic reticulum degradation) domain or Ubl4A-UBL to SGTA (**S5 Fig**; [116, 117]; reviewed in [118]). PF3D7_1313000, on the other hand, was also the only significant hit (with e-value of 6e-06) in the BLAST search using the amino acid sequences of *S*. *cerevisiae* Get5 (ScGet5) in *P*. *falciparum*. Although, we detected no peptides corresponding to PF3D7_1313000 in the BioID fraction, we still retained PF3D7_1313000 as a plausible parasite homolog of Bag6/Ubl4a/Get5. We reasoned that the overlapping hits between the BLAST output of Bag6, Ubl4A and ScGet5 were due to the shared ubiquitin-like domain feature and thus, we were unable to conclusively designate any one of them. It is also possible that multiple proteins play redundant roles as Get5/Bag6/Ubl4A in *P*. *falciparum* and further experiments are necessary to identify and characterize the plasmodial homolog.

**Fig 6:**
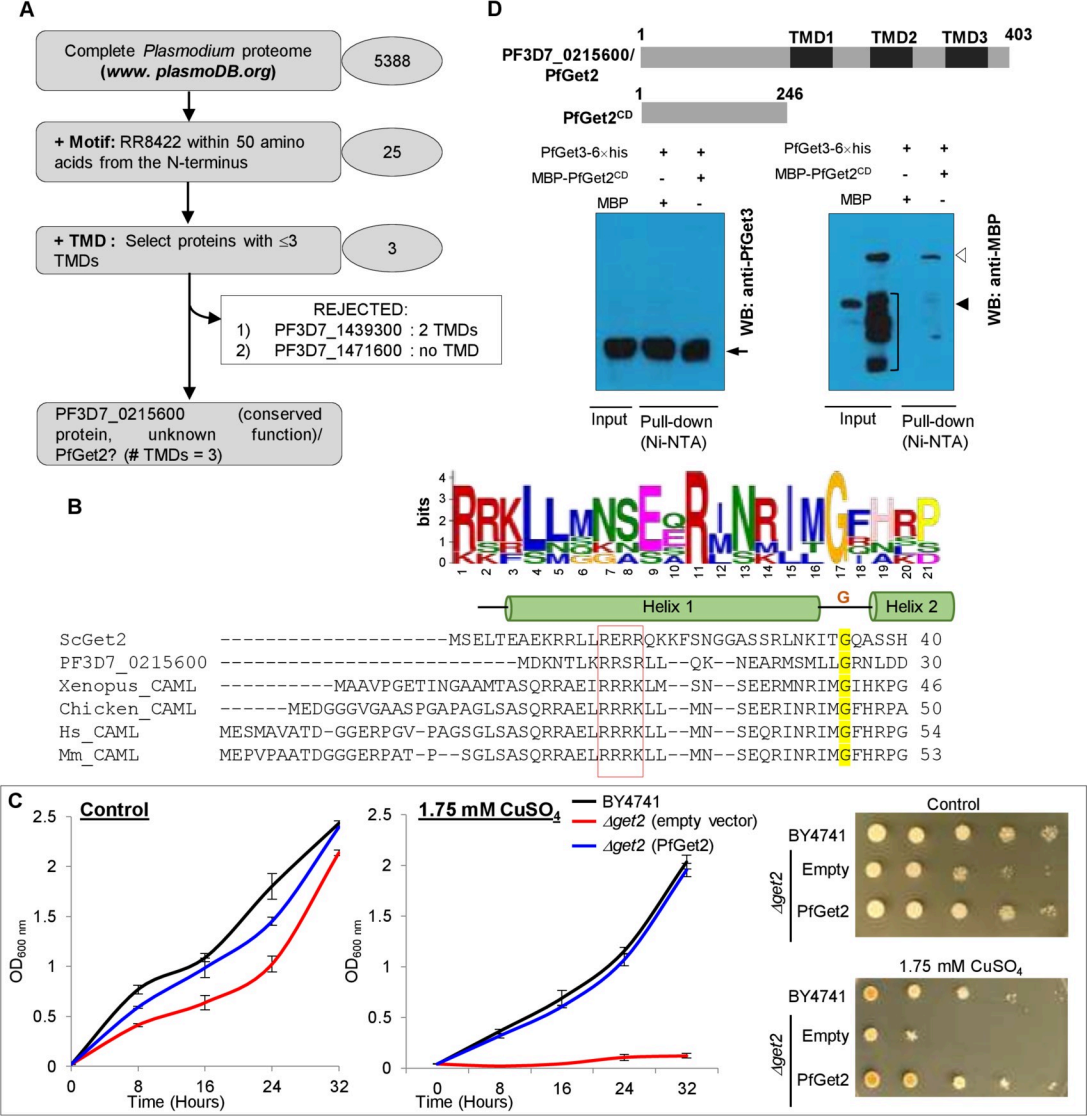
Identification of PfGet2 and *in vitro* association with PfGet3. **A.** Flow diagram showing the successive selection and elimination criteria in the bioinformatic prediction of PfGet2 in *P*. *falciparum* 3D7. **B.** Sequence alignments between the amino acids at the N-terminus of PF3D7_0215600/PfGet2, *S*. *cerevisiae* Get2 (ScGet2) and homologs of CAML by Clustal Omega (*https*:*//www*.*ebi*.*ac*.*uk/Tools/msa/*clustalo) and the representative logo generated by MEME suite (*https*:*//meme-suite*.*org/meme/tools/meme*). The conservation of the RERR motif in ScGet2 (implicated in interactions with the negatively charged residues of ScGet3 as in [122]) with PfGet2 and corresponding residues in CAML are boxed in red. The two helices connected by a flexible glycine linker (yellow highlighted). **C.** Growth curve of *S*. *cerevisiae* BY4741 wild-type (black) or *Δget2* cells transformed with either the empty vector (red) or with sequences encoding for PfGet2 (blue) grown in the absence (left) or the presence of 1.75 mM CuSO_4_ (right). While no growth differences are apparent in the absence of CuSO_4_, the salt-sensitive growth phenotype exhibited by *Δget2* cells transformed with the empty vector (red) is rescued by the expression of PfGet2 (blue). Corresponding images of the plate-based growth assay in the absence (top) or presence of 1.75 mM CuSO_4_ (bottom) are shown at the extreme right. **D.** Schematic of PfGet2 and PfGet2^CD^ (top). Bottom: western blots showing the association of the purified 6×his tagged recombinant PfGet3 (~43-kDa; black arrow) with MBP-PfGet2^CD^ (empty arrowhead) but not with MBP (empty region indicated by solid black arrowhead) as revealed by Ni^2+^-NTA beads pull-down assay followed by western blots using antibodies to MBP (right) or PfGet3 (left). Corresponding input and pull-down lanes are as indicated. Non-specific degradation products of MBP-PfGet2^CD^ are demarcated by square brackets (right).

The terminal step in TA translocation involves interaction of the TA-loaded Get3 with Get1/2 (or the mammalian WRB/CAML) receptors at the ER membrane to achieve membrane insertion, a process coupled with the ATP hydrolysis [119–121]. During this process, the positively charged residues at N-terminus of the ER-membrane anchored Get2/CAML are proposed to function as an antenna, firstly seeking to capture the TA complex and subsequently promoting its disassembly for insertion into the ER membranes, facilitated by the insertase activity of Get1/WRB [122, 123]. The C-terminus of CAML, on the other hand, has been implicated to play an important role as a membrane anchor as well as contribute towards the stability of Get1/WRB receptor [124, 125]. We were unable to identify any prospective plasmodial homolog Get2/CAML in our BioID fraction based on the amino acid sequence similarity/identity. However, cross-kingdom members of Get2/CAML have recently been shown to display extreme variations in sequences; rather the members exhibited conservation in their apparent structural properties, namely: a positively charged N-terminus stretch (with at least four arginine or lysine residues in a stretch), the putative numbers of TMDs (three) and the topology of the protein (N-terminus_Cytosol_-TMDs-C-terminus_Luminal_) [126]. We therefore mined the *P*. *falciparum* 3D7 proteome in PlasmoDB to shortlist proteins fulfilling both the criteria, *i*.*e*., (i) the presence of the RR8422 (Arg-Arg-Polar-Basic-Aliphatic-Aliphatic) motif (as a representation of the conserved RRRK motif commonly seen in CAMLs; [127]) within 50 amino acids from the N-terminus, and (ii) the presence of at least 3 TMDs (**Fig 6A**). Our results retrieved PFD_0215600 as the only hit. Interestingly, two peptides corresponding to PF3D7_0215600 were detected in the BioID fraction while none in the control (iBAQ fold enrichment >1000; see **Tables 2 and *S2***) thereby strongly indicating it as a potential homolog of Get2/CAML. In the PlasmoDB, PF3D7_0215600 is a 403 amino acid protein, without the ER-type SS and annotated as a conserved plasmodial protein of unknown function. It has three transmembrane regions spanning between 247–267, 299–322 and 348–368 amino acids and shared only 27% similarity and 14.4% identity with the *H*. *sapiens* CAML and 22.9% similarity and 12.4% identity with ScGet2 (**Fig 6B**). We have henceforth designated PF3D7_0215600 as PfGet2 in this study. PfGet2 also rescued the CuSO_4_-sensitive phenotype exhibited by the *Δget2* knockout stain of *S*. *cerevisiae* as compared to the vector-only transformed *Δget2* cells both in the liquid culture and plate-based growth assay (**Fig 6C**), thereby confirming its role as a putative homolog of ScGet2 in *P*. *falciparum*. The transmembrane topology prediction for PfGet2 by MEMSAT 3 [128] revealed that residues 1–246 are oriented towards the cytosol, we thus expressed and purified the recombinant cytosolic domain of PF3D7_0215600 (PfGet2^CD^; schematic shown in **Fig 6D**) fused to the MBP at the N-terminus (see *Materials and Methods*). Accordingly, purified MBP-PfGet2^CD^ or recombinant MBP (as a control) was incubated with the purified PfGet3-6×his, followed by enrichment with Ni-NTA beads and western blotting using anti- MBP or anti-PfGet3 antibodies (**Fig 6D**). Results revealed co-purification of MBP-PfGet2^CD^ (but not MBP) with recombinant PfGet3-6×his bound to Ni-NTA, thereby confirming a direct interaction. Thus, based on our bioinformatics selection parameters, enrichment in the BioID fraction and *in vitro* interactions results, we concluded that the *P*. *falciparum* genome encodes for a functional PfGet2 which exhibits properties similar to the other Get2 homologs.

**Fig 7:**
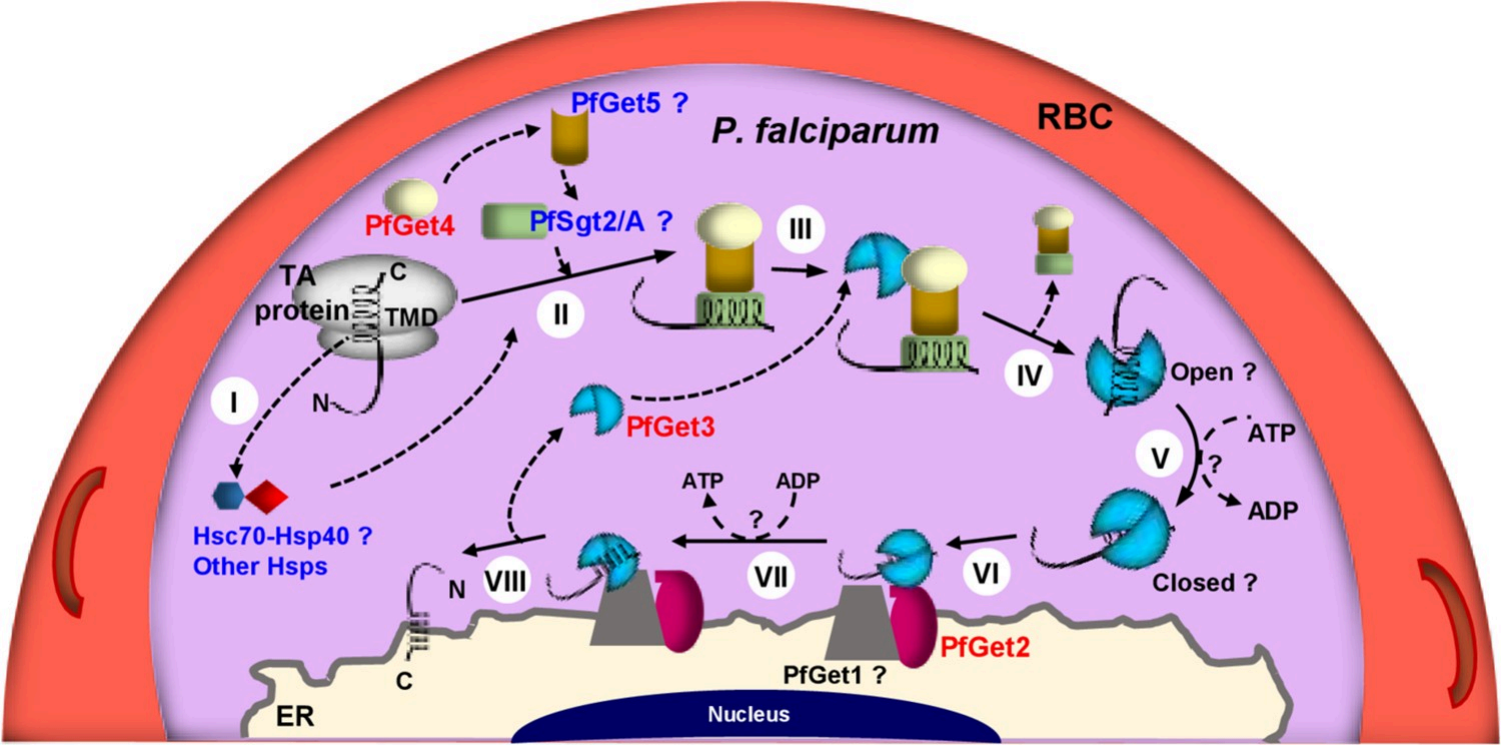
Schematic representation summarizing the predicted role(s) of the GET machinery homologs in the trafficking of *P*. *falciparum* TA-proteins. The ribosome emerging TMD of the translating TA protein either intermediately enlists the Heat shock (cognate) protein repertoire (Hs(c/p); **step I**), or the PfSgt2/A (in a complex with PfGet4 and PfGet5; **step II**) directly to sequester the hydrophobic TMD from the hydrophilic cytosol. PfGet3 then interacts with PfGet4 (**step III**) to enable the transfer of TA protein, binding of the TMD with PfGet3 and the release of PfSgt2/A-PfGet4-PfGet5 complex (**step IV**). PfGet3 may then shift from an open to a tighter closed conformation, dependent on ATP binding (**step V**). Subsequently, the TA-bound PfGet3 approaches the ER-membrane receptors, where it interacts first with PfGet2 (**step VI**), followed by an ATP-hydrolysis event (to revert to the loosely TA-bound open conformation) and concomitant binding to PfGet1 (**step VII**). Finally (step **VIII**), PfGet1 functions as an insertase to mediate the release of the TA protein from (now loosely bound) PfGet3 and insertion into the ER membrane with the N-terminus facing the parasite cytosol. The homologs of the GET pathway in *P*. *falciparum* validated or shortlisted in this study are represented in red or blue text, respectively. All unidentified/uncharacterized elements of the GET pathway in *P*. *falciparum* are indicated by question marks. ER, endoplasmic reticulum; RBC, red blood cell.

In conclusion, the outcome from this study revealed two novel aspects of protein trafficking in the human malaria parasite *P*. *falciparum*, namely the existence of TA proteins in the proteome, and the active role of GET machinery for the protein translocation onto destined organelles (**Fig 7**). A recent study identified 59 putative TA proteins encoded by the *Toxoplasma* genome [18]. Similarly, our bioinformatics predictions revealed a total of 63 TA proteins in the *P*. *falciparum*. We further validated these predictions in cellular assays using the BioID approach with PfGet3-mediated proximal labelling and showed that a subset of the TA repertoire is indeed associated with PfGet3, thus directly implicating the role of PfGet3 in their translocation. In addition, our in-depth mining, and analyses of mass spectrometric data from the PfGet3-BioID pool either precisely identified or shortlisted a few other homologs of the GET machinery in *P*. *falciparum*. These included PfGet4 and PfGet2. We were, however, only able to shortlist (and not conclusively identify) the candidate plasmodial homologs of some of the other GET components due to the omnipresence of the following conserved domains/sequence motifs across multiple hits in the BioID fraction: (i) TPR in Sgt2/A and, (ii) ubiquitin-like domain in Get5/UBL4A/Bag6. We were also unable to identify Get1/WRB in *P*. *falciparum*, possibly due to the extreme sequence divergence from the canonical Get1/WRB. Identification and validation of PfGet1 along with homologs of the plasmodial Get5/UBL4A/Bag6 forms the next challenge in our understanding of GET pathway in this parasite. Until then, we offer our results to the community to investigate further alongside us.

## Materials and methods

### Evolutionary analyses of PfGet3 and PfGet4 by Maximum Likelihood method

The evolutionary history of PfGet3 (PF3D7_0415000) and PfGet4 (PF3D7_1438600) were inferred by using the Maximum Likelihood method plus frequency model [104]. The bootstrap consensus tree inferred from 1000 replicates were taken to represent the evolutionary history of the taxa analyzed [129]. Branches corresponding to partitions reproduced in less than 50% bootstrap replicates were collapsed. The percentage of replicate trees in which the associated taxa clustered together in the bootstrap test (1000 replicates) have been shown next to the branches. Initial tree(s) for the heuristic search were obtained automatically by applying Neighbor-Join and BioNJ algorithms to a matrix of pairwise distances estimated using the JTT model, and then selecting the topology with superior log likelihood value. A discrete Gamma distribution was used to model evolutionary rate differences among sites (5 categories (+*G*, parameter = 4.1388 for PfGet3 and 66.6765 for PfGet4). The rate variation model allowed for some sites to be evolutionarily invariable ([+*I*], 0.46% sites) for PfGet4. The analysis involved 13 and 12 amino acid sequences for homologs of PfGet3 and PfGet4, respectively. There was a total of 409 (for PfGet3) or 327 (for PfGet4) positions in the final datasets. Evolutionary analyses were conducted in MEGA X [130].

### Plasmids and constructs

All plasmid constructs used for this study were generated using molecular biology grade reagents and verified by Sanger sequencing. For all the PCRs involving the amplification of plasmodial homologs of the GET pathway and sub-cloning into different plasmids, *P*. *falciparum* 3D7 genomic DNA was used as a template. Briefly, pET42b (PfGet3-6×his) was generated by PCR amplification of *pfget3* using the forward PfGet3-NdeIF (5’-AGATCTCATATGAGTGAG GATGAATCGAATTCCGTTTCTTGTTCATTAAGC-3’) and reverse PfGet3-XhoIR (5’-AGATCTCTCGAGCAGATTATCTTTATATATAGGTATATCTTTCGATTGTAAGAGCATCTCCG-3’) primers, digestion of the PCR product with NdeI-XhoI and cloning at corresponding sites within similarly digested pET42b. The yeast complementation plasmid pYES260 was purchased from Euroscarf (*http*:*//www*.*euroscarf*.*de/*) and contained *ura3* selectable marker [131]. For generating the plasmid pYES260 (PfGet3), *pfget3* was PCR amplified using PfGet3-NcoIF forward (5’-AGATCTCCATGGATAGTGAGGATGAATCGAATTCCG-3’) and PfGet3-XhoI reverse (5’-AGATCTGTCGACCTCGAGTTACAGATTATCTTTATATATAGGTATATCTTTCGATTG-3’) primers. The PCR product was digested with NcoI-XhoI and cloned at the corresponding sites in digested pYES260. Similar strategy was also used to generate pYES260 (PfGet4/PF3D7_1438600) and pYES260 (PfGet2/PF3D7_0215600) using the following primer pairs: PfGet4-HindIIIF (5’-CCCATAAAGCTTATGTATCCATATGATGTTCCAGATTATGCTGCAGCTGCTAAAAAGTTCAAATTTAGTAAAGAAAAGCTAGCC-3’)/ PfGet4-XhoISalIR (5’-AGATCTGTCGACCTCGAGTTATGCAAATATGTTTTGGAACATACTGAACAAGTTATG-3’) or PfGet2-HindIIIF (5’- CCCATAAAGCTTATGTATCCATATGATGTTCCAGATTATGCTGCAGCTGCTGATAAAAATACATTAAAAAGAAGAAG-3’)/ PfGet2FL-XhoIR (5’-AGA CCGCTCGAGTCATAATAATACTACCTTCTGG -3’). Cloning within these sites also ensured incorporation of sequences encoding for the HA-tag at the N-terminus of the recombinant PfGet4 in the *S*. *cerevisiae* transfectants.

The strategy was further extended to generate pYES260 constructs with genes encoding for the *S*. *cerevisiae* homologs of Get3 (ScGet3) and Get4 (ScGet4). Briefly, *scget3* and *scget4* were amplified by PCR from *S*. *cerevisiae* genomic DNA using the following primer pairs: ScGet3NcoIF (5’- CATGCCATGGATGATTTAACCGTGGAACCTAATTTG-3’)/ScGet3XhoIR (5’-AGATCTCTCGAGCTATTCCTTATCTTCTAACTCATAAATGACTTTG -3’) or ScGet4-NcoIF (5’-CATGCCATGGATTTCCTGCTGAATCTAATGCTGTACAAGC-3’)/ScGet4-XhoIR (5’-AGATCT CTCGAGTCACTTCGATCCGCCCAGGAAT-3’) and cloned at the NcoI and XhoI sites in pYES260.

The *P*. *falciparum* expression plasmid construct pA150 (PfGet3-GFP) was generated by PCR amplifying *pfget3* using PfGet3-AvrIIF (5’-GTACCGCCTAGGATGAGTGAGGATGAATCGAATTC-3’) and PfGet3-AAA-BglIIR (5’-TCCTTTAGATCTAGCTGCTGCCAGATTATCTTTATATATAGGTATATCTTTCGATTG-3’), restriction digestion and cloning into similarly digested pA150. For the BioID experiments, the plasmid pcDNA3.1 MCS-BirA(R118G)-HA was purchased from Addgene (USA) and used to construct pA150 (PfGet3- BirA*-HA) through a series of subcloning steps. Briefly, *pfget3* was PCR amplified using PfGet3-AvrIIF and PfGet3-AAA-BglIIR primers, first cloned at NheI-BamHI sites of pcDNA3.1 MCS-BirA(R118G)-HA. Subsequently, this plasmid used as template to PCR amplify out the entire *pfget3-birA (R118G)-ha* fragment using PfGet3-AvrIIF and HA-XhoIR (5’-TCTAGACTCGAGCTATGCGTAATCCGGTACATCGTAAG-3’) primers, digested with AvrII-XhoI and subcloned into similarly digested pA150. The construct pA150 (HA-PfSec61b) was generated by amplifying *pfsec61b/pf3d7_0821800* from *P*. *falciparum* genomic DNA using PCR with the forward HA-PfSec61b-AvrIIF (5’-CCCATAAAGCTTCCTAGGATGTATCCATATGATGTTCCAGATTATGCTGCAGCTGCTAACGCCGCACCAGTAATAG-3’) and PfSec61b-XhoIR (5’-AGATCTCTCGAGTATTTTACTAATGATATGTAAAATAACTACACTTGCCATAAATATTAATG-3’) primers, digestion with AvrII-XhoI and cloning into similarly digested pA150.

The constructs for the expression of N-terminus MBP tagged recombinant PfGet4 or PfGet2^CD^, *i*.*e*., pMALc2X (PfGet4) or pMALc2x (PfGet2^CD^) were generated by amplification of either full-length *pfget4* or nucleotides encoding for the first 246 amino acids of PfGet2^CD^ using PCR with the PfGet4-EcoRIF (5’-GTACCGGAATTCATGAAAAAGTTCAAATTTAGTAAAGAAAAGCTAGCC-3’)/PfGet4-XhoISalIR or PfGet2CD-BamHIF (5’-GTACGCGGATCCATGGATAAAAATACATTAAAAAGAA-3’)/PfGet2CD-XhoISalIR (5’-AGACCGGTCGACCTCGAGTTATTCATGTTTCGTAATAATAAATTG-3’) primer pairs and subsequently cloning at corresponding sites in pMALc2x plasmid (New England Biolabs). For the co-expression and co-association studies in *E*. *coli*, *pf3d7_1111300/bos1* and *pf3d70710800/use1* were amplified by PCR using *P*. *falciparum* 3D7 genomic DNA and the respective primer pairs: BOS1-BamHIF (5’-GATCGGATCCTATGAATAAAAAGTATGAACCAATATTAGATAATGAATTG-3’)/ BOS1-AgeISalXhoIR (5’-CTCGAGGTCGACACCGGTTCATCTCTTAAAGTAAGAATATATGACATAAAAAAATATC-3’) and USE1-BamHIF (5’-GATCGGATCCTATG ATTGTGTTCGACGAACTTTTTG-3’)/ USE1-AgeISaIlXhoIR (5’-CTCGAGGTCGACACCGGTTTATAAAAGTATTATTACAAAAAAGGTAAATATAAAAAGTACTACTGAAG-3’). The PCR products were digested with BamHI and SalI and cloned in the MCS-1 of pCDFDuet-1 (Novagen, Merck, Germany). This resulted in *in-frame* fusion at the 5’ end with sequences in the vector encoding for the 6×his tag at the N-terminus of the BOS1 and USE1 fusion proteins. Subsequently, *pfget3* was subcloned from pET42b (PfGet3) construct at the NdeI-XhoI sites at the MCS-2, which generated *in-frame* fusion with at the 3’ end with nucleotides encoding for the S-tag at the C-terminus of the PfGet3 fusion protein.

### Expression and purification of recombinant PfGet3-6×his, MBP, MBP-PfGet4 and MBP-PfGet2^CD^ from *E*. *coli*

*E*. *coli* BL21 arabinose-inducible (AI) cells (Invitrogen) were transformed with the plasmids pET42b (PfGet3-6×his), pMALc2x (PfGet4) or pMALc2x (PfGet2^CD^) and transformants were selected on 100 μg/ml ampicillin or 25 μg/ml kanamycin containing LB plates. Primary cultures of transformed *E*. *coli* cells were grown overnight at 37°C under shaking conditions and then used to seed secondary cultures. Cultures were induced with 1 mM isopropyl β-D-1-thiogalactopyranoside (IPTG) and 0.2% L-Arabinose at OD_600_ ≈ 0.6 for 16 h at 25°C. For the purification of PfGet3-6×his, induced cultures were harvested at 6,000 rpm for 20 mins and resuspended in lysis buffer (50 mM Tris. HCl pH 8.0, 500 mM NaCl, 10 mM imidazole,1 mM MgCl_2_, 5mM β-mercaptoethanol, 5% glycerol) containing 100 mM Phenylmethylsulphonylfluoride (PMSF) and EDTA-free Protease inhibitor cocktail tablets (Roche). Resuspended cells were first treated with 1 mg/ml lysozyme to digest the cell wall and subsequently frozen at -80°C overnight. The thawed cell suspension was sonicated at 30% amplitude with repetitive pulses of 30 s and off for 30 s till clarity. The sonicated cells were centrifuged at 14,000 rpm for 1 h at 4°C to remove the cell debris and the supernatant was incubated to Ni^2+^-NTA resin (Thermo Fisher) pre equilibrated with lysis buffer. After binding 4°C for 4 h under rotating conditions, the supernatant-resin suspension was packed onto a column and washed with at least 20 column volumes of wash buffer (lysis buffer containing 40 mM imidazole). PfGet3-6×his was eluted by elution buffer (lysis buffer containing 300 mM imidazole) and the eluted fractions were analyzed by running SDS-PAGE. Fractions containing PfGet3-6×his were pooled, dialyzed against 10 mM Tris. HCl pH 8.0, 150 mM NaCl, 1 mM MgCl_2_, 5 mM β-mercaptoethanol and 5% glycerol with minimum of three changes in buffer and concentrated using membrane concentrators (10-kDa MWCO; Millipore). The purified recombinant PfGet3-6×his was subsequently used for the custom generation of anti-PfGet3 antibodies or for *in vitro* binding experiments.

For the purification of recombinant MBP, MBP-PfGet4 and MBP-PfGet2^CD^, the respective secondary cultures (OD_600_ ≈ 0.6) of transformed AI cells were induced with 0.3 mM IPTG at 25°C for 16 h, harvested at 6,000 rpm for 20 min and resuspended in column buffer (20 mM Tris. HCl, pH 7.4, 200 mM NaCl, 1 mM EDTA and 10 mM β-mercaptoethanol). Soluble supernatant fractions were generated following similar protocol as described above. MBP, MBP-PfGet4 and MBP-PfGet2^CD^ were affinity purified using amylose-resin (Thermo Fisher, USA) and eluted with elution buffer (50 mM Tris. HCl pH 8.0, 200 mM NaCl, 1 mM EDTA, 5 mM β-mercaptoethanol and 10 mM maltose) as according to manufacturer’s instructions. The corresponding proteins were dialysed against 10 mM Tris. HCl pH 8.0, 150 mM NaCl, 1 mM MgCl_2_, 5 mM β-mercaptoethanol and concentrated. The purity of each protein was assessed by SDS-PAGE and subsequently used for *in vitro* binding studies.

### Generation of custom antibodies against PfGet3 and PfGet4

Antibodies were generated against PfGet3 and PfGet4. Anti-PfGet3 antibodies were custom-generated in rabbits by Biobharati LifeScience (India) using the purified recombinant PfGet3-6×his as immunogen. Following booster, the anti-PfGet3 antibodies were affinity purified from harvested sera using column bound recombinant PfGet3-6×his. For anti-PfGet4 antibodies, the KLH-conjugated peptide CPPNSTEEKYKFMN, corresponding to the amino acids 97–110 in PfGet4 (PF3D7_1438600), was used as an immunogen in New Zealand rabbits and custom-generated by Genscript (USA). Antibodies were also affinity purified against the same peptide.

### Transformation of *S*. *cerevisiae* BY4741 wild type and knockout strains

*S*. *cerevisiae* parental BY4741 (S288C isogenic yeast strain: *MATa; his3D1; leu2D0; met15D0; ura3D0*), *Δget3* (BY4741; *MATa; ura3Δ0; leu2Δ0; his3Δ1; met15Δ0; YDL100c*::*kanMX4*),*Δget4* (BY4741; *MATa; ura3Δ0; leu2Δ0; his3Δ1; met15Δ0; YOR164c*::*kanMX4*) or *Δget2* (BY4741; *MATa; his3Δ1; leu2Δ0; met15Δ0; ura3Δ0; YER083c*::*kanMX4*) strains were purchased from Euroscarf (*http*:*//www*.*euroscarf*.*de*). The constructs pYES260 (PfGet3), pYES260 (PfGet4), pYES260 (PfGet2) or empty plasmid pYES260 were used to transform the respective strains. All the yeast transformations were performed following the previously published protocol [132] and selected in SD media lacking uracil. After transformation, the cells were grown overnight in Yeast Nitrogen Base (YNB) Sc-Ura media at 30°C under shaking conditions. To verify the expression of recombinant PfGet3 or PfGet4 in transformed *S*. *cerevisiae*, cells were inoculated in YNB Sc-Ura media and grown. The cell density was subsequently adjusted to OD_600 nm_ ≈ 0.01 and induced with 2.5% galactose and 1% raffinose for 8 h following which cells were harvested at 6,000 rpm for 5 min at 25°C and washed with autoclaved milliQ. Cells were then resuspended in lysis buffer (50 mM Tris.HCl, pH 7.5, 1% sodium deoxycholate; 1% Triton X-100; 0.1% SDS; 50 mM sodium fluoride; 0.1 mM sodium vanadate; 5 mM sodium pyrophosphate) containing 0.05% PMSF and EDTA-free Complete protease inhibitor cocktail (Roche). Equal volume of acid washed 0.45 mm glass beads (Sigma) were added to the cells and homogenized using BeadBeater (BioSpec) at 3,000 rpm for 30 s followed by 30 s on ice for a total of 8 cycles. Cell suspensions were decanted away from the glass beads and centrifuged twice at 10,000 rpm at 4°C to remove unlysed cells and cellular debris. Supernatants were quantified and analyzed by western blotting using anti-PfGet3 or anti-PfGet4 antibodies.

### Quantitative growth assay of parental BY4741 and *Δget3* or *Δget4* transformants

The *S*. *cerevisiae* parental BY4741 strain or the *Δget2*/*Δget3*/*Δget4* knockouts transformed with either the empty pYES260 plasmids or expressing the plasmodial homologs were grown overnight in YNB Sc-Ura at 30°C under shaking conditions at 200 rpm. After growth, the respective cell density was adjusted to OD_600 nm_ ≈ 0.01, cells transferred to 50 ml falcon tube containing fresh 15 ml YNB SC-Ura and induced with 2.5% galactose and 1% raffinose in the presence or absence of 1.5 mM or 1.75 mM CuSO_4_. Cells were grown for 32 h at 30°C under shaking conditions during which samples were harvested at every 8 h time interval to measure OD_600 nm_ and the growth curve was plotted accordingly. For the plate-based growth assay, induced cultures were normalized to OD_600 nm_ ≈ 1.0, serially diluted five-fold in sterile autoclaved water and 5 μl of each of the 10^−1^ to 10^−5^ dilutions were spotted onto YNB only or YNB with CuSO_4_ plates. Plates were then incubated at 30°C for 36 h before analysis.

### *P*. *falciparum* parasite cultures, generation of transgenic parasites and microscopy

*P*. *falciparum* 3D7 parasites were grown in fresh O+ human RBCs (kindly provided by the Rotary Blood Bank) in RPMI 1640 media supplemented with 0.5% Albumax II, 0.2 mM hypoxanthine, 11 mM glucose, 5% NaHCO_3_ and 50 μg/ml gentamycin in a humidified CO_2_ incubator as described previously [48]. Cultures were monitored daily by Giemsa staining of methanol-fixed smears and fed as necessary. The parasitemia was generally maintained below 12% for healthy culture growth. Transgenic parasites were generated by electroporation of plasmid DNA according to previously published protocols [133]. For live cell imaging of transgenic parasites expressing PfGet3-GFP as well as for IFA, the parasite nucleus was stained with Hoechst 33342, processed as described previously [134] and imaged with a 100× NA objective on a Zeiss ApoTome 2 (Axiovert 40 CFL) under fluorescence and brightfield optics.

### Indirect Immunofluorescence assay (IFA) of *P*. *falciparum* parasites

Indirect immunofluorescence assays (IFA) were performed with either non-transfected *P*. *falciparum* 3D7 or transgenic parasites following protocol as described previously [135]. Briefly, fixed smears of parasites were fixed with 4% paraformaldehyde/0.0075% glutaraldehyde and permeabilized with 0.1% Triton X-100. The free aldehyde group was neutralized with 50 mM NH_4_Cl, and slides blocked with 2% BSA. The respective primary antibodies were diluted in blocking buffer and incubated at RT for 2 h. The following antibody concentrations were used: anti-PfGet3, 10 μg/ml; anti-Exp2 ([133]), 20 μg/ml; anti-BiP ([133]), 10 μg/ml; anti-PfGet4, 10 μg/ml; anti-HA, 10 μg/ml; anti-PfGet3, 1:200 and anti-plasmepsin V (MRA-815A, MR4), 1:200 dilution. The corresponding FITC- or TRITC-labelled secondary IgG antibodies (ICN Biochemicals) were used at 1:200 dilution. The parasite nuclei were stained with 5 μg/ml Hoechst 33342 (Molecular Probes) and slides were mounted with DABCO. The slides were viewed as described previously.

### SDS-PAGE and western blotting

The preparation of lysates from transformed bacterial and yeast cultures are described previously. For the preparation *P*. *falciparum* parasites, cultures are 10–12% parasitemia were permeabilized with PBS (137 mM NaCl, 2.7 mM KCl,10 mM Na_2_HPO_4_, 2 mM KH_2_PO_4_, pH 7.4) containing 0.05% saponin to release the hemoglobin content of the host red blood cell. The parasite fraction was repeatedly washed (at 3,200 rpm for 10 min) with ice cold PBS till no hemoglobin was detected in the supernatant fraction. The parasite pellet was subsequently solubilised in Laemmli’s sample buffer containing 1 mM dithiothreitol (DTT), denatured for 10 min at 95°C and resolved by 12% SDS-PAGE. Resolved proteins were then transferred onto a nitrocellulose membrane and blocked with 5% fat-free skim milk for 2 h. Membranes were incubated with either anti-PfGet3, anti-Hog (kindly provided by Prof. A. Mondal, SLS, JNU), anti-GFP (Invitrogen) or anti-β actin antibodies for 3 h on a platform shaker and followed by respective HRP-conjugated secondary antibodies. The blots were finally developed by chemiluminescence detection (Amersham).

### Proximity labelling (BioID) experiments

Proximity biotinylation experiment was performed with the transgenic *P*. *falciparum* 3D7 parasites expressing PfGet3-BirA*-HA following previously published protocol [136]. Briefly, 140 ml of asynchronous cultures were split equally into two 175 cm^2^ flasks at 2% parasitemia and 5% hematocrit. D-Biotin was added at a final concentration of 50 μg/ml to one of the flasks (designated as the BioID sample) and not to the other (representing the control). The parasites were allowed to grow for 48 h at 37°C with replacement of fresh media (± D-Biotin) after 24 h. Cultures were subsequently harvested, washed twice with cold PBS and RBCs were lysed in PBS containing 0.03% saponin for 10 min. The parasite pellet was washed at least five times with 10 volumes of cold PBS to remove all the hemoglobin and stored at -80°C till further use. When planned, the frozen parasites were thawed at 4°C and extracted for 1 h at 4°C with 2 ml lysis buffer (50 mM Tris. HCl pH 7.5, 500 mM NaCl, 1% Triton-X-100) containing 1 mM DTT, 1 mM PMSF and protease inhibitor cocktail (Roche) under rotating conditions. The soluble extract was separated from insoluble cellular debris by centrifugation at 16,000 × g for 30 min at 4°C and immediately diluted two-fold with 50 mM Tris. HCl, pH 7.5. An aliquot each of the BioID and control extracts was stored at -80°C for SDS-PAGE and western blotting. To the remaining extracts, 30 μl of StrepTactin XT was added and allowed to incubate overnight under rotating condition at 4°C. After incubation, the beads were pelleted by centrifugation at 3,000 rpm for 5 min at 4°C and washed sequentially: thrice with lysis buffer, twice with cold double distilled water and twice with 50 mM Tris. HCl, pH 7.5. The beads were finally boiled in SDS-PAGE sample buffer and processed either for western blotting using anti-PfGet3 or anti-HA, or for LC-MS/MS analyses.

### Mass Spectrometry analyses and peptide quantitation

Mass spectrometry of the control and BioID fractions were performed at Wistar Proteomics Facility, Philadelphia, PA, USA. Briefly, the fractions were resolved by SDS-PAGE (only to the extent when the samples entered 0.5 cm within the resolving gel) and stained with colloidal Coomassie. Bands were excised from the gel, de-stained with water, alkylated with iodoacetamide, and digested with trypsin as previously described [48] (see S1 Text). Protein quantification was performed using Razor + unique peptides. Razor peptides were the shared (non-unique) peptides assigned to the protein group with the most other peptides (Occam’s razor principle). MS/MS count referred to how many times peptides belonging to the protein were sequenced and Intensity denoted the sum of the peptide MS peak areas for the protein. The iBAQ (Intensity Based Absolute Quantification) values represented protein intensity divided by the number of theoretical peptides and are roughly considered to be proportional to the molar quantities of the proteins.

### Co-association and pulldown studies with recombinant PfGet3 and candidate TA proteins

For co-expression and co-association studies, *E*. *coli* Rosetta™ (DE3) cells (Novagen, Merck, Germany) were transformed with pCDFDuet plasmids, cloned with *pfget3* along with either *pf3d7_1111300/bos1* or *pf3d70710800/use1*. As a control, pCDFDuet expressing PfGet3 alone was used. Transformants were selected in LB agar plates with spectinomycin and inoculated into terrific broth containing the desired antibiotic. Cultures and grown at 37°C under shaking conditions, induced with 0.5 mM IPTG at OD_600 nm_ of 0.6–0.8 for 3–4 h at 37°C. Cells were harvested prior to and after induction by centrifugation at 6,000 × g for 20 mins at 4°C. The induction of specific proteins was confirmed in western blots prior to processing for pulldown experiments.

For the downstream pulldown protocols using Ni^2+^-NTA resin, the harvested cells pellets (post-induction) were lysed by lysis buffer (50 mM Tris. HCl pH 8, 500 mM NaCl, 20 mM imidazole, 5 mM β-mercaptoethanol, 100 μM PMSF (phenylmethylsulphonyl fluoride), 1 mg/ml lysozyme and protease inhibitor tablets cocktail tablets (Roche, Switzerland). The cell suspensions were disrupted by sonication with cycles of 30 seconds on and off each at 30% amplitude, for approximately 5 minutes till the turbidity of the solutions were clarified. The sonicated cells were centrifuged at 12,000 rpm for 30 mins at 4°C to remove the insoluble cellular debris and the soluble lysates were incubated to Ni^2+^-NTA resin (Thermo Fisher, USA) pre-equilibrated with the lysis buffer for 2 h at 4°C under gentle rotating conditions. Following binding, the resin-lysate suspensions were packed onto column and extensively washed with wash buffer (50 mM Tris.HCl pH 8, 500 mM NaCl, 40 mM imidazole, 5 mM β-mercaptoethanol). The proteins were stripped off the beads by boiling in 2× SDS PAGE sample buffer for 5 min at 95°C, resolved SDS-PAGE and processed for western blotting using anti-PfGet3 and anti-His (Biobharati Lifesciences, India) antibodies.

### *In vitro* binding studies with recombinant PfGet3-6×his, MBP, MBP-PfGet4 and MBP-PfGet2^CD^

For *in vitro* binding studies, purified recombinant MBP-PfGet4 or MBP-PfGet2^CD^ (2–5 μg) were incubated with recombinant PfGet3-6×his in 1 mg/ml BSA containing binding buffer (50 mm Tris. HCl, pH 8.0, 200 mM NaCl, 0.1% NP-40, 20 mM Imidazole and 1 mm DTT) for 2 h at 4°C. As a control, PfGet3-6×his was also incubated with purified MBP at similar concentration and duration. Following binding, binding buffer-equilibrated Ni^2+^-NTA beads were added to all protein mixtures and allowed to bind at 4°C for 2 h under shaking conditions. Beads were then washed extensively with binding buffer (without BSA), boiled in the presence of SDS-PAGE sample buffer for 5 min at 95°C and resolved by SDS-PAGE. Nitrocellulose membrane-transferred proteins were incubated with anti-PfGet3 or anti-MBP (NEB), probed with corresponding HRP-conjugated secondary antibodies and developed by chemiluminescence.

## Supporting information

S1 Fig Percentage identity and similarity of PfGet3 with the other homologs of Get3. **A.** Clustal X sequence alignment [141] between PfGet3 and the putative homologs: *S*. *cerevisiae*, *D*. *hansenii*, *S*. *pombe*, *A*. *fumigatus*, *H*. *sapiens*, *A*. *thaliana* and the bacterial Arsenite transporter ArsA. Residue colouring is based on the program output (type of amino acid). The shading of the bars from brown to yellow reflects the degree of conservation, quality, and the consensus amino acids of the ordinates. Occupancy at a particular residue position is indicated by increasing intensity from light to dark grey shading. **B.** Table summarizing the similarity and identity between the amino acid sequences of PfGet3 (PF3D7_0415000) in comparison to the other validated or predicted homologs of Get3.
(TIFF)

S2 Fig Secondary structure prediction for PfGet3. **A.** Predicted secondary structure of PfGet3 by the Phyre2 server (*www*.*sbg*.*bio*.*ic*.*ac*.*uk/phyre2*) and revealing the presence of 51% α-helices, 10% β-strands and 21% disordered regions. Residues are colored according to a simple property-based scheme: A, S, T, G and P; small/polar are in yellow, M, I, L and V; hydrophobic are in green, K, R, E, N, D, H and Q; charged are in red, and W, Y, F, C; aromatic + cysteine are in purple. The secondary structure prediction comprises three states: α-helix, β-strand, or coil. Green helices represent α-helices, blue arrows indicate β-strands and faint lines indicate coils. The ‘SS confidence’ line indicates the confidence in the prediction from PSIPRED, with red indicating high confidence and blue showing low confidence. A large amount of blue or green in the confidence line is indicative of few homologous sequences detected and a consequent low probability of modeling success. **B.** Outcome of the TMD prediction for PfGet3 by the TMHMM server (*www*.*cbs*.*dtu*.*dk/services/TMHMM-2*.*0*). No transmembrane helix was predicted in PfGet3.
(TIFF)

S3 Fig Expression of recombinant PfGet3-6×his in *E*. *coli*. SDS PAGE (left) and western blot (middle and right) showing the expression of recombinant 6×his tagged PfGet3 in *E*. *coli* cells only under IPTG induced conditions, as compared to the uninduced control. The induced recombinant PfGet3 is indicated as filled arrowhead in the SDS PAGE (left) or by empty arrowheads in the western blots (middle and right) using custom-generated antibodies to PfGet3 (middle) or commercial anti-6×his antibodies (Biobharati Lifesciences, India). Molecular weight standards (in kDa) are as indicated.
(TIF)

S4 Fig Sequence alignments of a few representative homologs of Get4 and the predicted secondary structure of PfGet4. **A.** Table showing the percentage identity and similarity between the various homologs of Get4 in comparison to PfGet4. **B.** Result from the secondary structure prediction for PfGet4 by the Phyre2 server (*www*.*sbg*.*bio*.*ic*.*ac*.*uk/phyre2*) and revealing the presence of 72% α-helices and 14% disordered regions. Residues are colored according to a simple property-based scheme: A, S, T, G and P; small/polar are in yellow, M, I, L and V; hydrophobic are in green, K, R, E, N, D, H and Q; charged are in red, and W, Y, F, C; aromatic + cysteine are in purple. The secondary structure prediction comprises three states: α-helix, β-strand, or coil. Green helices represent α-helices, blue arrows indicate β-strands and faint lines indicate coils. The ‘SS confidence’ line indicates the confidence in the prediction from PSIPRED, with red indicating high confidence and blue showing low confidence. A large amount of blue or green in the confidence line is indicative of few homologous sequences detected and a consequent low probability of modeling success. **C.** Multiple sequence alignment between PfGet4 and a few representative homologs of Get4 using ClustalX [141]. Residue colouring is based on the program output (type of amino acid). The shading of the bars from brown to yellow reflects the conservation number, quality, and consensus amino acids of the ordinates. Occupancy at a particular residue position is indicated by increasing intensity of light to dark grey shading. **D.** Phyre2 predicted 3D structure of PfGet4 (rainbow colored) aligned with the crystal structure of the *H*. *sapiens* TRC35 (PDB ID 6AU8A; grey) [102]. The α- helices are numbered and the N- and C-terminal domains are as indicated.
(TIFF)

S5 Fig Sequence alignment showing conservation of residues between Bag6-UBL, Ubl4A-UBL and the potential plasmodial homologs. NMR chemical shift perturbation patterns of Ubl4A-UBL and Bag6-UBL caused by their corresponding interaction partners (N-terminus of SGTA for Ubl4A-UBL and CUE for Bag6-UBL are indicated by asterisks [116]. Black triangles indicate chemical shift perturbations which only occur significantly to Bag6-UBL. Open triangles indicate significant chemical perturbations that only occur to residues in Ubl4A-UBL. Only the UBL domains of PF3D7_0922100, PF3D7_1313000 and PF3D7_1211800 were selected for the alignment.
(TIF)

S1 Table List of the total 130 predicted TA proteins, the 67 misrepresented TA proteins and the final shortlisted 63 predicted TA proteins in the *P*. *falciparum* 3D7 proteome. The list of 130 proteins includes RIFINs (64), EVP1, REX-2 and MSP5. The PlasmoDB ID, description and other features are shown for each predicted TA protein. Corresponding GRAVY and Adagir scores are also shown and shaded according to the scale provided. Intracellular localization is also predicted for each TA protein based on three different machine learning tools (LOCKTREE 3, BUSCA and DeepLoc 1.0) and reveals no clear consensus for any particular organelle. Thus, the 63 predicted TA proteins were manually grouped (in this study) into three predicted categories based on their Gene Ontology (GO) annotations in the Uniprot database (*http*:*//uniprot*.*org*): ER (shaded light orange), mitochondria (grey) and other destinations (not shaded). Thus, a total of 14 ER-specific TA proteins, 7 mitochondrial-specific TAs and 42 TAs with diverse cellular destinations were predicted.
(XLSX)

S2 Table Raw data of the LC-MS/MS analyses of the BioID and control fractions. (XLSX)

S1 Text Prediction algorithms used in the bioinformatic determination of TA proteins in *P. falciparum*. (DOCX)
