# Supplementary figures and images for: A conserved guided entry of tail-anchored pathway is involved in the trafficking of a subset of membrane proteins in Plasmodium falciparum
Source: PLoS Pathog. 2021 Nov 15;17(11):e1009595. doi: 10.1371/journal.ppat.1009595 (PMC8629386; doi:10.1371/journal.ppat.1009595)

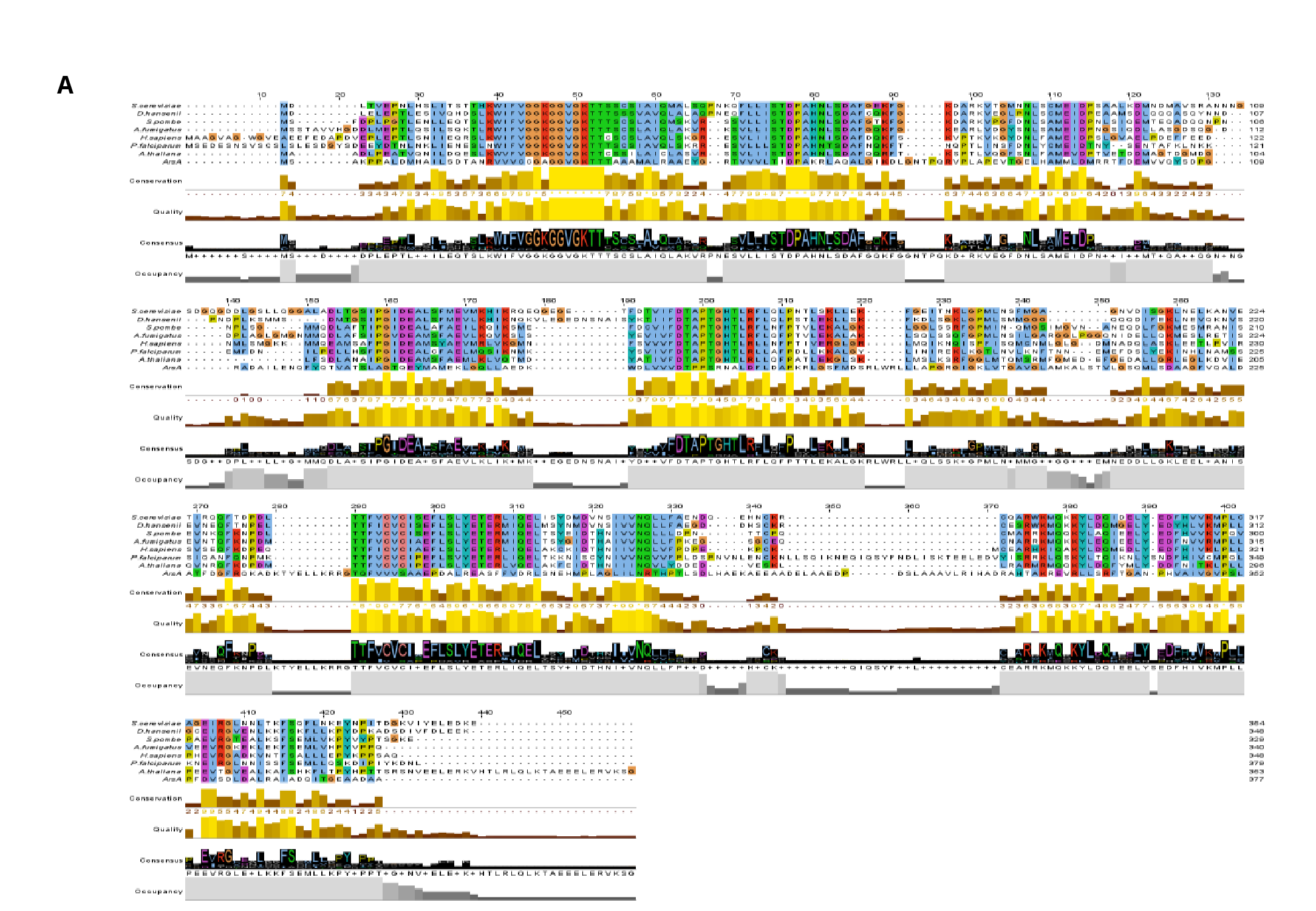

Supplement: S1 Fig — A. Clustal X sequence alignment [141] between PfGet3 and the putative homologs: S. cerevisiae, D. hansenii, S. pombe, A. fumigatus, H. sapiens, A. thaliana and the bacterial Arsenite transporter ArsA. Residue colouring is based on the program output (type of amino acid). The shading of the bars from brown to yellow reflects the degree of conservation, quality, and the consensus amino acids of the ordinates. Occupancy at a particular residue position is indicated by increasing intensity from light to dark grey shading. B. Table summarizing the similarity and identity between the amino acid sequences of PfGet3 (PF3D7_0415000) in comparison to the other validated or predicted homologs of Get3. (TIFF) [file ppat.1009595.s001.tiff]

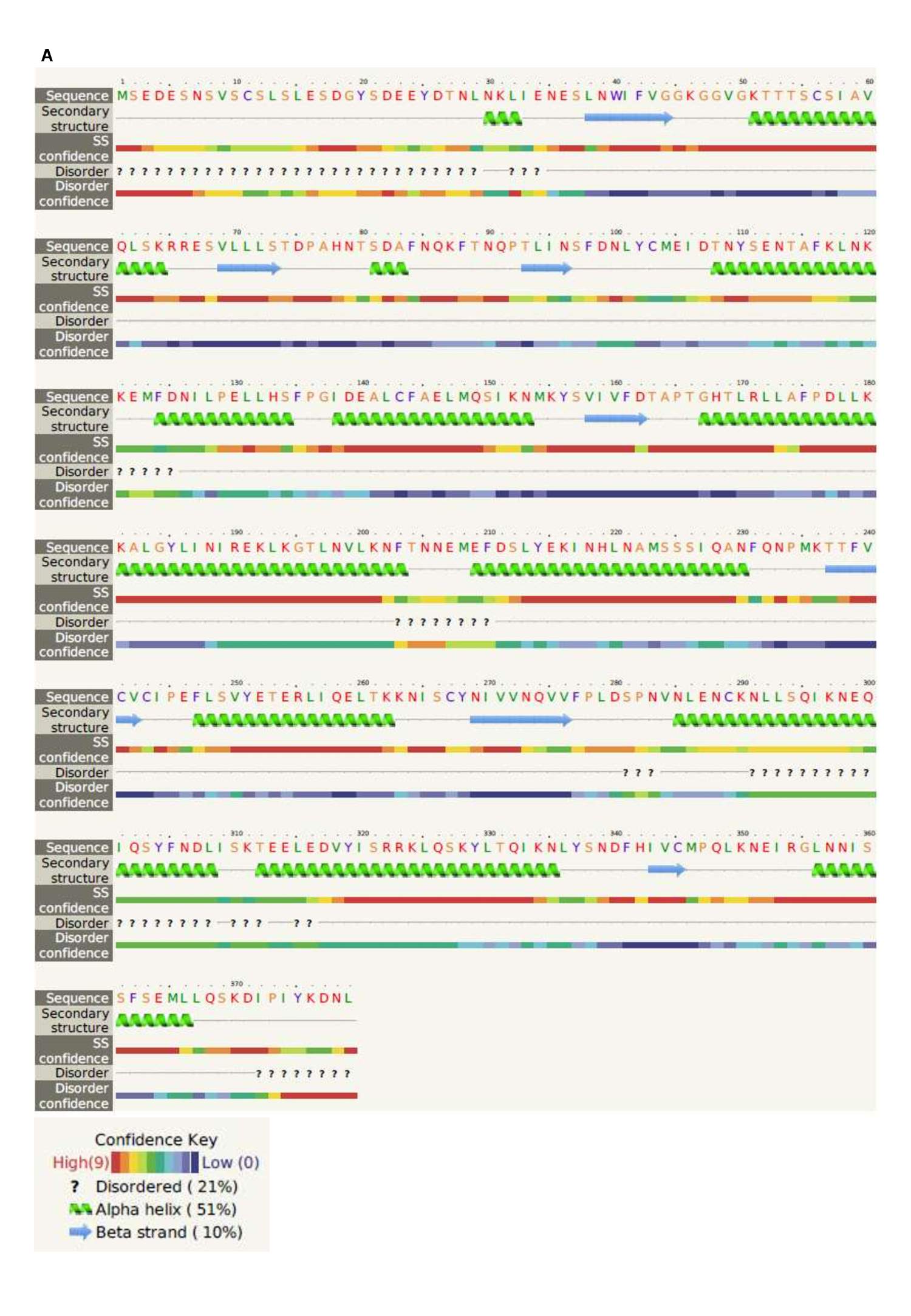

Supplement: S2 Fig — A. Predicted secondary structure of PfGet3 by the Phyre2 server (www.sbg.bio.ic.ac.uk/phyre2) and revealing the presence of 51% α-helices, 10% β-strands and 21% disordered regions. Residues are colored according to a simple property-based scheme: A, S, T, G and P; small/polar are in yellow, M, I, L and V; hydrophobic are in green, K, R, E, N, D, H and Q; charged are in red, and W, Y, F, C; aromatic + cysteine are in purple. The secondary structure prediction comprises three states: α-helix, β-strand, or coil. Green helices represent α-helices, blue arrows indicate β-strands and faint lines indicate coils. The ‘SS confidence’ line indicates the confidence in the prediction from PSIPRED, with red indicating high confidence and blue showing low confidence. A large amount of blue or green in the confidence line is indicative of few homologous sequences detected and a consequent low probability of modeling success. B. Outcome of the TMD prediction for PfGet3 by the TMHMM server (www.cbs.dtu.dk/services/TMHMM-2.0). No transmembrane helix was predicted in PfGet3. (TIFF) [file ppat.1009595.s002.tiff]

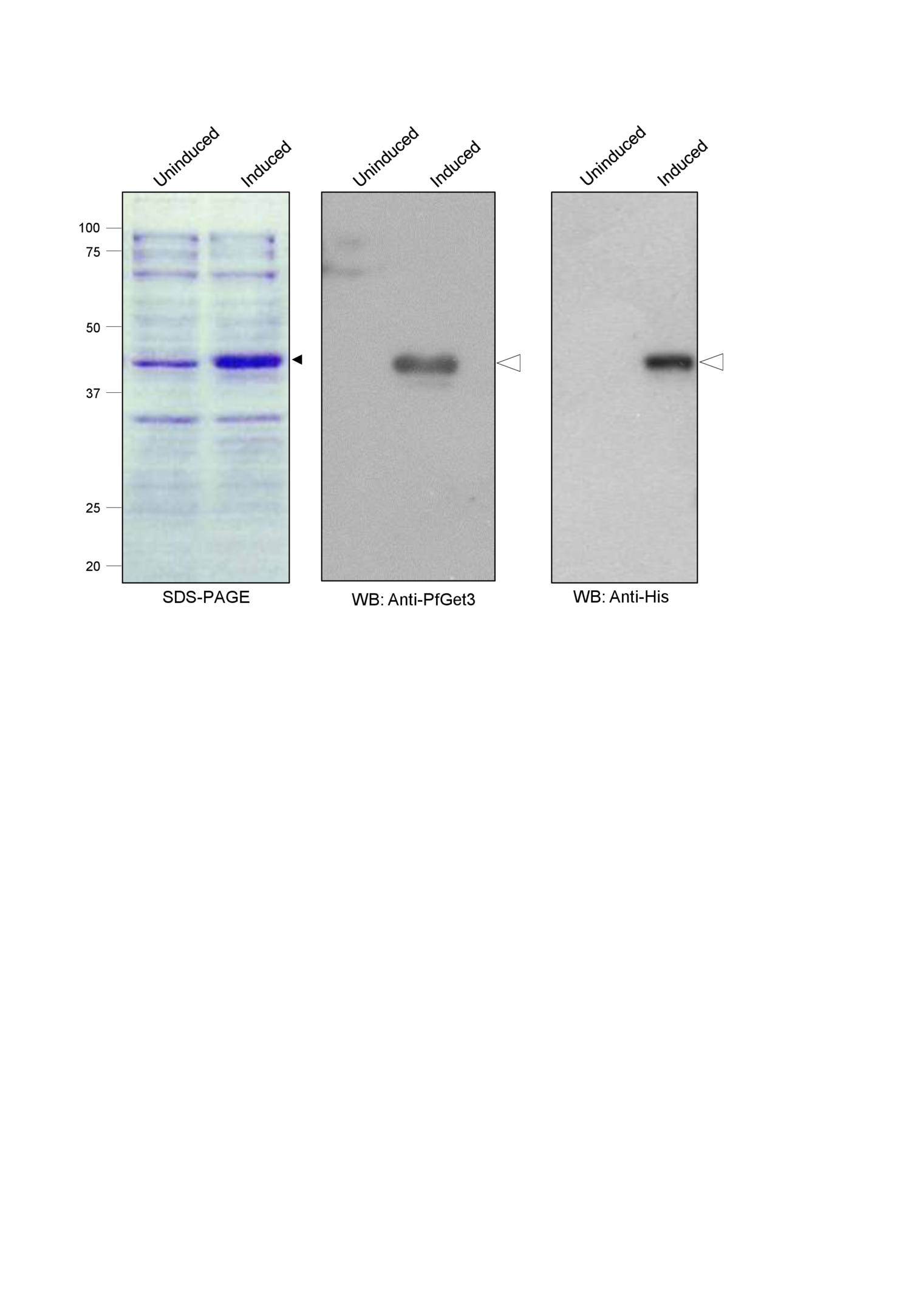

Supplement: S3 Fig — SDS PAGE (left) and western blot (middle and right) showing the expression of recombinant 6×his tagged PfGet3 in E. coli cells only under IPTG induced conditions, as compared to the uninduced control. The induced recombinant PfGet3 is indicated as filled arrowhead in the SDS PAGE (left) or by empty arrowheads in the western blots (middle and right) using custom-generated antibodies to PfGet3 (middle) or commercial anti-6×his antibodies (Biobharati Lifesciences, India). Molecular weight standards (in kDa) are as indicated. (TIF) [file ppat.1009595.s003.tif]

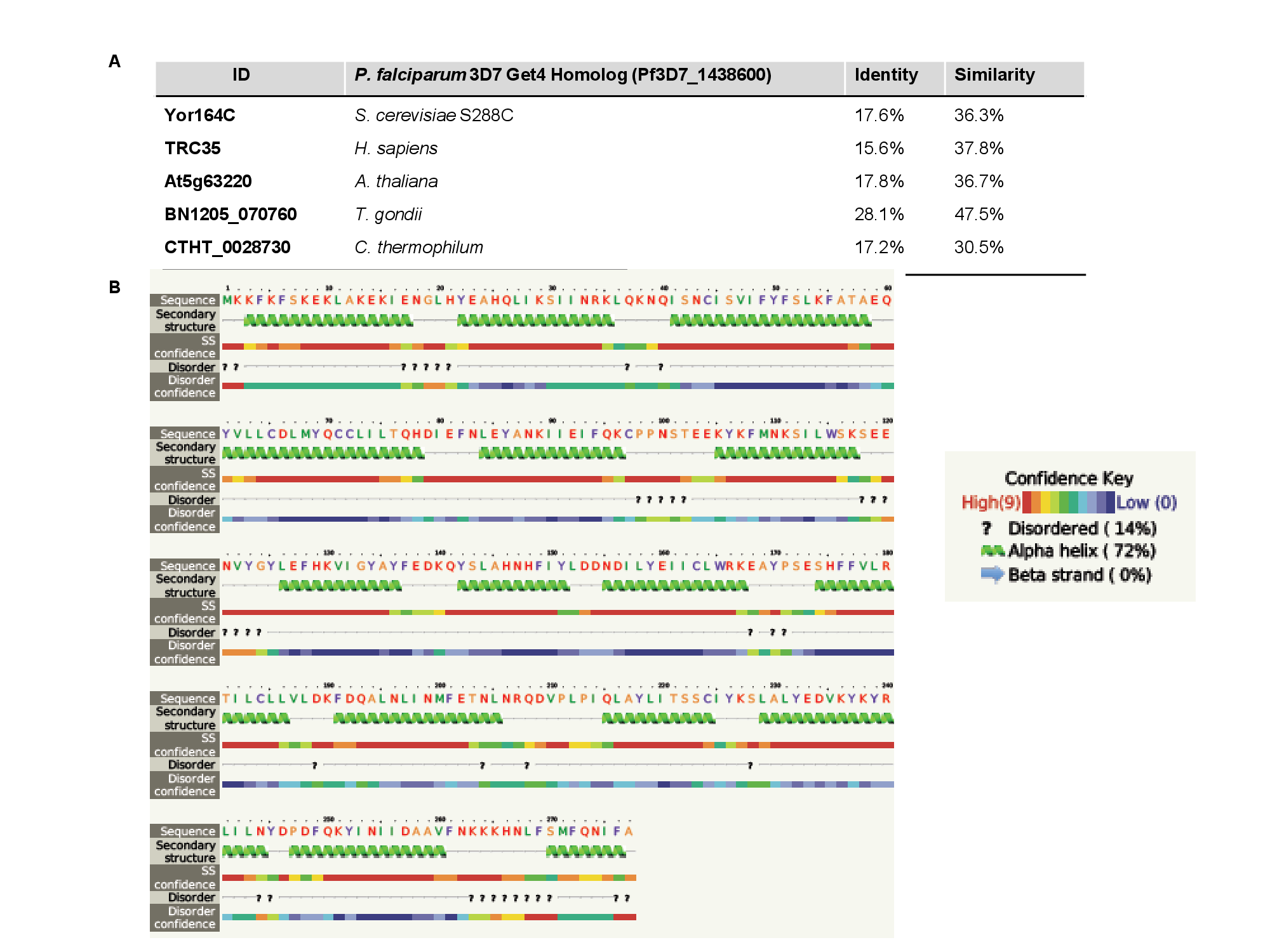

Supplement: S4 Fig — A. Table showing the percentage identity and similarity between the various homologs of Get4 in comparison to PfGet4. B. Result from the secondary structure prediction for PfGet4 by the Phyre2 server (www.sbg.bio.ic.ac.uk/phyre2) and revealing the presence of 72% α-helices and 14% disordered regions. Residues are colored according to a simple property-based scheme: A, S, T, G and P; small/polar are in yellow, M, I, L and V; hydrophobic are in green, K, R, E, N, D, H and Q; charged are in red, and W, Y, F, C; aromatic + cysteine are in purple. The secondary structure prediction comprises three states: α-helix, β-strand, or coil. Green helices represent α-helices, blue arrows indicate β-strands and faint lines indicate coils. The ‘SS confidence’ line indicates the confidence in the prediction from PSIPRED, with red indicating high confidence and blue showing low confidence. A large amount of blue or green in the confidence line is indicative of few homologous sequences detected and a consequent low probability of modeling success. C. Multiple sequence alignment between PfGet4 and a few representative homologs of Get4 using ClustalX [141]. Residue colouring is based on the program output (type of amino acid). The shading of the bars from brown to yellow reflects the conservation number, quality, and consensus amino acids of the ordinates. Occupancy at a particular residue position is indicated by increasing intensity of light to dark grey shading. D. Phyre2 predicted 3D structure of PfGet4 (rainbow colored) aligned with the crystal structure of the H. sapiens TRC35 (PDB ID 6AU8A; grey) [102]. The α- helices are numbered and the N- and C-terminal domains are as indicated. (TIFF) [file ppat.1009595.s004.tiff]

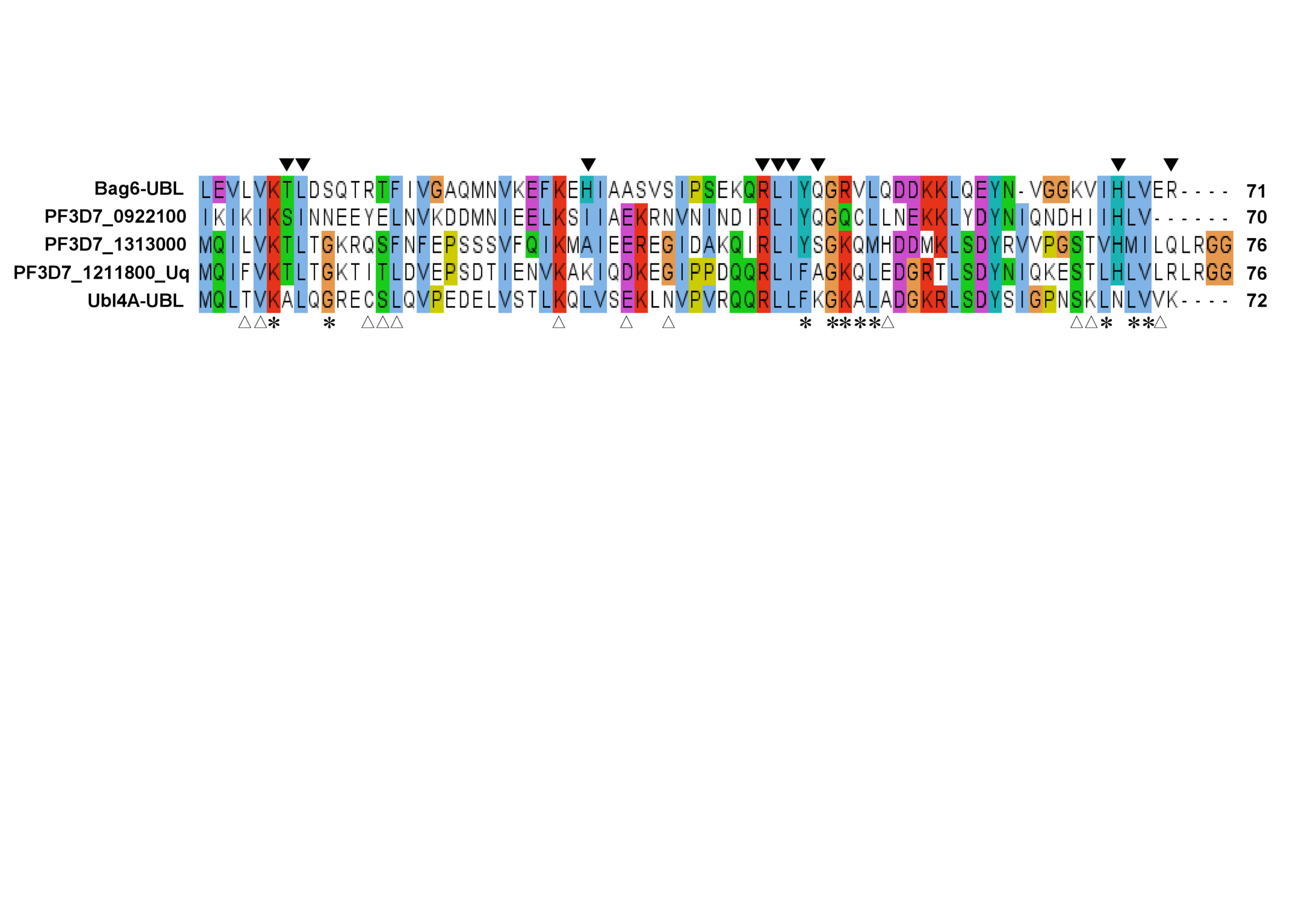

Supplement: S5 Fig — NMR chemical shift perturbation patterns of Ubl4A-UBL and Bag6-UBL caused by their corresponding interaction partners (N-terminus of SGTA for Ubl4A-UBL and CUE for Bag6-UBL are indicated by asterisks [116]. Black triangles indicate chemical shift perturbations which only occur significantly to Bag6-UBL. Open triangles indicate significant chemical perturbations that only occur to residues in Ubl4A-UBL. Only the UBL domains of PF3D7_0922100, PF3D7_1313000 and PF3D7_1211800 were selected for the alignment. (TIF) [file ppat.1009595.s005.tif]
