# Supplementary material for: A conserved guided entry of tail-anchored pathway is involved in the trafficking of a subset of membrane proteins in Plasmodium falciparum
Source: PLoS Pathog. 2021 Nov 15;17(11):e1009595. doi: 10.1371/journal.ppat.1009595 (PMC8629386; doi:10.1371/journal.ppat.1009595)
Supplement: S1 Text — (DOCX) [file ppat.1009595.s008.docx]

**S1 Text**

**Prediction algorithms used in the bioinformatic determination of TA proteins in *P. falciparum***

The Grand Average of Hydropathy (GRAVY) (*http://www.gravy-calculator.de*) score assigns hydrophobicity indices to the TMDs based on Kyte and Doolittle [1], while the Adagir scores (*http://agadir.crg.es*) reflect the relative tendency to form helices in aqueous solution.

Since there are no available machine learning tools to predict the subcellular localization of proteins exclusively dedicated to apicomplexan parasites including *Plasmodium*, the three subcellular localization predicting algorithms were considered, namely LOCTREE3 (*https://rostlab.org/services/loctree3*) [2], BUSCA (Bologna Unified Subcellular Component Annotator) (*http://busca.biocomp.unibo.it*) [3] and DeepLoc version 1.0 (*http://www.cbs.dtu.dk/services/DeepLoc*) [4]. All portrayed notable discrepancies in their outputs for the plasmodial proteins. Moreover, anaerobic protists and some parasites including *Plasmodium* display absence of peroxins and peroxisomes due to evolutionary reduction [5], thus ruling out this organelle. Further, *Plasmodium* parasites have unique organelles such as the apicoplast, food vacuole, rhoptries and micronemes which are likely to contain TA proteins but not represented in any algorithm. For example, apicoplast is reminiscent of chloroplast in plants, and chloroplasts contain essential TA proteins like SECE1 and SECE2 [6].

**Mass spectrometry analyses and peptide quantitation**

The digests were analyzed by LC-MS/MS using reverse phase capillary HPLC with a 75 µm nano-column interfaced on-line with a Thermo Electron LTQ OrbiTrap XL mass spectrometer. The mass spectrometer was operated in data dependent mode with full scans performed in the Orbitrap and parallel MS/MS analysis of the six most intense precursor ions in the linear ion trap. MS data were searched with full tryptic specificity against the UniProt *P. falciparum* 3D7 database and a common contaminant database using MaxQuant 1.6.17.0. The custom database consisted of a combination of the expected sequences with the various mutations of the proteins used in this experiment, the *E. coli* proteome (to provide a reasonable sized database), and expected common contaminants including keratins, trypsin, etc. A reversed sequence database was appended to the front of the forward database and used to estimate peptide false positive rates. Data were searched using partial tryptic specificity, a maximum of three missed cleavages, mass tolerance of 100 ppm, cysteine fixed as the carboxymethyl derivative, and dynamic methionine oxidation and N-terminal acetylation. Resulting data was filtered on 5 ppm and dCn of 0.07. The false positive rate for peptide identifications using these database search and data filtering parameters was set at 1%. Variable modification search included Biotinylation (+226.0776) on Lysines, Glycine-Glycine (+114.0429) on Lysines, *i.e*., tryptic remnant of ubiquitinylation or neddylation, acetylation (+42.01056) on protein N-terminus and oxidation (+15.99491) on Methionines. Fixed modifications searched include carbamidomethylation (+57.02146) on Cysteines. Protein quantification was performed using Razor + unique peptides. Razor peptides were the shared (non-unique) peptides assigned to the protein group with the most other peptides (Occam's razor principle). MS/MS count referred to how many times peptides belonging to the protein were sequenced and Intensity denoted the sum of the peptide MS peak areas for the protein. The iBAQ (Intensity Based Absolute Quantification) values represented protein intensity divided by the number of theoretical peptides and are roughly considered to be proportional to the molar quantities of the proteins.

**Legends to supporting figures and tables**

**S1 Fig. Percentage identity and similarity of PfGet3 with the other homologs of Get3. A.** Clustal X sequence alignment [145] between PfGet3 and the putative homologs: *S. cerevisiae*, *D. hansenii*, *S. pombe*, *A. fumigatus*, *H. sapiens*, *A. thaliana* and the bacterial Arsenite transporter ArsA. Residue colouring is based on the program output (type of amino acid). The shading of the bars from brown to yellow reflects the degree of conservation, quality, and the consensus amino acids of the ordinates. Occupancy at a particular residue position is indicated by increasing intensity from light to dark grey shading. **B.** Table summarizing the similarity and identity between the amino acid sequences of PfGet3 (PF3D7_0415000) in comparison to the other validated or predicted homologs of Get3.

**S2 Fig. Secondary structure prediction for PfGet3.** **A.** Predicted secondary structure of PfGet3 by the Phyre2 server (*www.sbg.bio.ic.ac.uk/phyre2*) and revealing the presence of 51% α-helices, 10% β-strands and 21% disordered regions. Residues are colored according to a simple property-based scheme: A, S, T, G and P; small/polar are in yellow, M, I, L and V; hydrophobic are in green, K, R, E, N, D, H and Q; charged are in red, and W, Y, F, C; aromatic + cysteine are in purple. The secondary structure prediction comprises three states: α-helix, β-strand, or coil. Green helices represent α-helices, blue arrows indicate β-strands and faint lines indicate coils. The ‘SS confidence’ line indicates the confidence in the prediction from PSIPRED, with red indicating high confidence and blue showing low confidence. A large amount of blue or green in the confidence line is indicative of few homologous sequences detected and a consequent low probability of modeling success. **B.** Outcome of the TMD prediction for PfGet3 by the TMHMM server (*www.cbs.dtu.dk/services/TMHMM-2.0*). No transmembrane helix was predicted in PfGet3.

**S3 Fig. Expression of recombinant PfGet3-6×his in *E. coli*.** SDS PAGE (left) and western blot (middle and right) showing the expression of recombinant 6×his tagged PfGet3 in *E. coli* cells only under IPTG induced conditions, as compared to the uninduced control. The induced recombinant PfGet3 is indicated as filled arrowhead in the SDS PAGE (left) or by empty arrowheads in the western blots (middle and right) using custom-generated antibodies to PfGet3 (middle) or commercial anti-6×his antibodies (Biobharati Lifesciences, India). Molecular weight standards (in kDa) are as indicated.

**S4 Fig. Sequence alignments of a few representative homologs of Get4 and the predicted secondary structure of PfGet4.** **A.** Table showing the percentage identity and similarity between the various homologs of Get4 in comparison to PfGet4. **B.** Result from the secondary structure prediction for PfGet4 by the Phyre2 server (*www.sbg.bio.ic.ac.uk/phyre2*) and revealing the presence of 72% α-helices and 14% disordered regions. Residues are colored according to a simple property-based scheme: A, S, T, G and P; small/polar are in yellow, M, I, L and V; hydrophobic are in green, K, R, E, N, D, H and Q; charged are in red, and W, Y, F, C; aromatic + cysteine are in purple. The secondary structure prediction comprises three states: α-helix, β-strand, or coil. Green helices represent α-helices, blue arrows indicate β-strands and faint lines indicate coils. The ‘SS confidence’ line indicates the confidence in the prediction from PSIPRED, with red indicating high confidence and blue showing low confidence. A large amount of blue or green in the confidence line is indicative of few homologous sequences detected and a consequent low probability of modeling success. **C.** Multiple sequence alignment between PfGet4 and a few representative homologs of Get4 using ClustalX [145]. Residue colouring is based on the program output (type of amino acid). The shading of the bars from brown to yellow reflects the conservation number, quality, and consensus amino acids of the ordinates. Occupancy at a particular residue position is indicated by increasing intensity of light to dark grey shading. **D.** Phyre2 predicted 3D structure of PfGet4 (rainbow colored) aligned with the crystal structure of the *H. sapiens* TRC35 (PDB ID 6AU8A; grey) [103]. The α- helices are numbered and the N- and C-terminal domains are as indicated.

**S5 Fig. Sequence alignment showing conservation of residues between Bag6-UBL, Ubl4A-UBL and the potential plasmodial homologs**. NMR chemical shift perturbation patterns of Ubl4A-UBL and Bag6-UBL caused by their corresponding interaction partners (N-terminus of SGTA for Ubl4A-UBL and CUE for Bag6-UBL are indicated by asterisks [118]. Black triangles indicate chemical shift perturbations which only occur significantly to Bag6-UBL. Open triangles indicate significant chemical perturbations that only occur to residues in Ubl4A-UBL. Only the UBL domains of PF3D7_0922100, PF3D7_1313000 and PF3D7_1211800 were selected for the alignment.

**S1 Table. List of the total 130 predicted TA proteins, the 67 misrepresented TA proteins and the final shortlisted 63 predicted TA proteins in the *P. falciparum* 3D7 proteome.** The list of 130 proteins includes RIFINs (64), EVP1, REX-2 and MSP5. The PlasmoDB ID, description and other features are shown for each predicted TA protein. Corresponding GRAVY and Adagir scores are also shown and shaded according to the scale provided. Intracellular localization is also predicted for each TA protein based on three different machine learning tools (LOCKTREE 3, BUSCA and DeepLoc 1.0) and reveals no clear consensus for any particular organelle. Thus, the 63 predicted TA proteins were manually grouped (in this study) into three predicted categories based on their Gene Ontology (GO) annotations in the Uniprot database (*http://uniprot.org*): ER (shaded light orange), mitochondria (grey) and other destinations (not shaded). Thus, a total of 14 ER-specific TA proteins, 7 mitochondrial-specific TAs and 42 TAs with diverse cellular destinations were predicted.

**S2 Table. Raw data of the LC-MS/MS analyses of the BioID and control fractions.**

**References**

1. Kyte J, Doolittle RF. A simple method for displaying the hydropathic character of a protein. Journal of Molecular Biology. 1982/05/05. 1982;157: 105–132. doi:10.1016/0022-2836(82)90515-0
2. Goldberg T, Hecht M, Hamp T, Karl T, Yachdav G, Ahmed N, et al. LocTree3 prediction of localization. Nucleic Acids Research. 2014;42. doi:10.1093/nar/gku396
3. Savojardo C, Martelli PL, Fariselli P, Profiti G, Casadio R. BUSCA: An integrative web server to predict subcellular localization of proteins. Nucleic Acids Research. 2018;46. doi:10.1093/nar/gky320
4. Almagro Armenteros JJ, Sønderby CK, Sønderby SK, Nielsen H, Winther O. DeepLoc: prediction of protein subcellular localization using deep learning. Bioinformatics (Oxford, England). 2017;33. doi:10.1093/bioinformatics/btx431
5. Ludewig Klingner AK, Michael M, Jarek M, Brinkmann H, Petersen J. Distribution and Evolution of Peroxisomes in Alveolates (Apicomplexa, Dinoflagellates, Ciliates). Genome Biology and Evolution. 2018;10: 1–13. doi:10.1093/gbe/evx250.
